# Unveiling microbial communities with EasyAmplicon: A user‐centric guide to perform amplicon sequencing data analysis

**DOI:** 10.1002/imo2.42

**Published:** 2024-11-20

**Authors:** Salsabeel Yousuf, Hao Luo, Meiyin Zeng, Lei Chen, Tengfei Ma, Xiaofang Li, Maosheng Zheng, Xin Zhou, Liang Chen, Jiao Xi, Hongye Lu, Huiluo Cao, Xiaoya Ma, Bian Bian, Pengfan Zhang, Jiqiu Wu, Renyou Gan, Baolei Jia, Linyang Sun, Zhicheng Ju, Yunyun Gao, Waqar Afzal Malik, Chuang Ma, Hujie Lyu, Yahui Li, Huiyu Hou, Yuanping Zhou, Defeng Bai, Yao Wang, Haifei Yang, Jiani Xun, Shengda Du, Tianyuan Zhang, Xiulin Wan, Kai Peng, Shanshan Xu, Tao Wen, Tong Chen, Yong‐Xin Liu

**Affiliations:** ^1^ Genome Analysis Laboratory of the Ministry of Agriculture and Rural Affairs, Agricultural Genomics Institute at Shenzhen, Chinese Academy of Agricultural Sciences Shenzhen Guangdong China; ^2^ Department of Vascular Surgery, Fu Xing Hospital Capital Medical University Beijing China; ^3^ State Key Laboratory of Herbage Improvement and Grassland Agro‐Ecosystems, Centre for Grassland Microbiome, College of Pastoral Agriculture Science and Technology Lanzhou University Lanzhou Gansu China; ^4^ Centre for Agricultural Resources Research, Institute of Genetics and Developmental Biology, Chinese Academy of Sciences Shijiazhuang Hebei China; ^5^ College of Environmental Science and Engineering North China Electric Power University Beijing China; ^6^ State Key Laboratory of Mycology, Institute of Microbiology, Chinese Academy of Sciences Beijing China; ^7^ Biomedical Innovation Center and Beijing Key Laboratory for Therapeutic Cancer Vaccines, Beijing Shijitan Hospital Capital Medical University Beijing China; ^8^ College of Natural Resources and Environment Northwest A&F University Yangling Shaanxi China; ^9^ Stomatology Hospital, School of Stomatology Zhejiang University School of Medicine, Zhejiang Provincial Clinical Research Center for Oral Diseases, Key Laboratory of Oral Biomedical Research of Zhejiang Province, Cancer Center of Zhejiang University, Engineering Research Center of Oral Biomaterials and Devices of Zhejiang Province Hangzhou Zhejiang China; ^10^ Department of Microbiology University of Hong Kong Pok Fu Lam Hong Kong China; ^11^ Center for Energy Metabolism and Reproduction, Institute of Biomedicine and Biotechnology, Shenzhen Institute of Advanced Technology, Chinese Academy of Sciences Shenzhen Guangdong China; ^12^ Department of Cardiology, Shenzhen Guangming District People's Hospital Shenzhen Guangdong China; ^13^ Department of Computational Biology and Medical Sciences, Graduate School of Frontier Sciences The University of Tokyo Kashiwa, Chiba Japan; ^14^ Innovative Genomics Institute University of California Berkeley California USA; ^15^ APC Microbiome Institute University College Cork Cork Ireland; ^16^ Department of Genetics University Medical Center Groningen, University of Groningen Groningen The Netherlands; ^17^ Department of Food Science and Nutrition, Faculty of Science The Hong Kong Polytechnic University Kowloon Hong Kong China; ^18^ Xianghu Laboratory Hangzhou Zhejiang China; ^19^ Faculty of Biological & Environmental Sciences University of Helsinki Helsinki Finland; ^20^ Department of Ocean Science The Hong Kong University of Science and Technology Clear Water Bay Hongkong China; ^21^ School of Horticulture Anhui Agricultural University Hefei Anhui China; ^22^ Department of Life Sciences, Imperial College of London London UK; ^23^ Guangdong Provincial Key Laboratory of Medical Molecular Diagnostics, The First Dongguan Affiliated Hospital, College of Basic Medicine Guangdong Medical University Dongguan Guangdong China; ^24^ College of Life Sciences Qingdao Agricultural University Qingdao Shandong China; ^25^ College of Life Sciences Northwest A&F University Yangling Shaanxi China; ^26^ Guangdong Hybribio Biotech Co., Ltd. Chaozhou Guangdong China; ^27^ Jiangsu Co‐Innovation Center for Prevention and Control of Important Animal Infectious Diseases and Zoonoses, College of Veterinary Medicine Yangzhou University Yangzhou Jiangsu China; ^28^ School of Food and Biological Engineering Hefei University of Technology Hefei Anhui China; ^29^ Jiangsu Provincial Key Lab for Solid Organic Waste Utilization, Key Lab of Organic‐Based Fertilizers of China, Jiangsu Collaborative Innovation Center for Solid Organic Wastes, Educational Ministry Engineering Center of Resource‐Saving Fertilizers Nanjing Agricultural University Nanjing Jiangsu China; ^30^ State Key Laboratory for Quality Assurance and Sustainable Use of Dao‐di Herbs, National Resource Center for Chinese Materia Medica, China Academy of Chinese Medical Sciences Beijing China

**Keywords:** computational biology, EasyAmplicon, metagenome, microbiome

## Abstract

The advent of next‐generation sequencing has revolutionized microbiome research, enabling in‐depth exploration of microbial communities through amplicon sequencing. The widespread adoption of sequencing across diverse fields, coupled with decreasing costs, underscores the critical need for validated, fully automated, reproducible, and adaptable analysis pipelines. However, analyzing these high‐throughput datasets often necessitates extensive bioinformatics expertize, hindering accessibility for many researchers. To address this challenge, in 2023 we developed EasyAmplicon, a comprehensive, user‐friendly pipeline that integrates popular tools such as USEARCH and VSEARCH, offering a streamlined workflow from raw data to results. Remarkably, EasyAmplicon has garnered significant recognition within a year, as evidenced by 127 citations to date. To further facilitate the researchers and enhance usability, we present a detailed protocol with a video recording that guides users through each step of the pipeline, including data preprocessing (quality filtering, chimera removal), amplicon sequence variant analysis, diversity analysis, and data visualization. The protocol is designed for ease of use, with each step documented, allowing researchers to execute the workflow without requiring complex scripting skills. The EasyAmplicon pipeline is freely available on GitHub (https://github.com/YongxinLiu/EasyAmplicon).

## Highlights

- EasyAmplicon offers a fully automated, user‐friendly pipeline for comprehensive amplicon sequencing analysis, seamlessly integrating various bioinformatics tools.
- The pipeline supports efficient data preprocessing, diversity analysis, and advanced visualization, making high‐throughput data analysis accessible to a broader range of researchers.
- Each step is documented, along with troubleshooting tips and solutions, for users' convenience.

## Graphical abstract

The EasyAmplicon pipeline workflow for amplicon sequencing analysis begins with sample collection and next‐generation sequencing, which generates sequencing data alongside crucial metadata for contextual understanding. This user‐friendly pipeline is compatible with Windows, Linux, and macOS, offering both command‐line and interactive modes in RStudio. The workflow consists of three main steps: dimensionality reduction, analysis, and statistics & visualization, which generate standard inputs for tools like STAMP and LEfSe. Key analyses include α and β diversity, taxonomic composition, and differential biomarkers, emphasizing EasyAmplicon capability to effectively analyzing microbial communities through the integration of sequencing data and metadata.

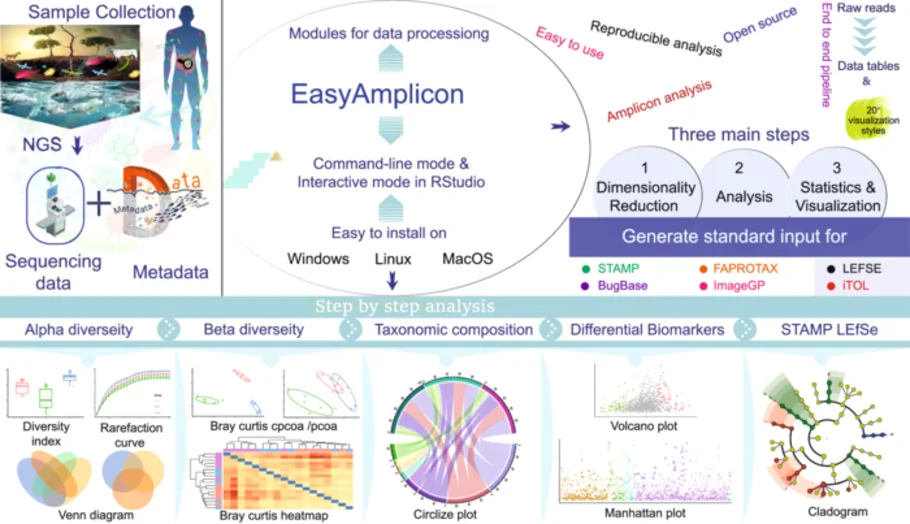

## 1 INTRODUCTION

The technology of next‐generation sequencing (NGS) is widely employed to describe microbial communities in various environments [1, 2, 3], exemplified by initiatives such as the Human Microbiome Project (https://www.hmpdacc.org/hmp/index.php) and the Earth Microbiome Project (https://earthmicrobiome.org/) [4, 5, 6, 7]. Over the past two decades, advances in NGS technology [8, 9], data analysis methods, and bioinformatics tools have significantly enhanced our understanding of the crucial roles played by microbiomes in humans [10], animals [11], plants [12] and the environment [13], as well as the interactions among them [14]. These advances are also applied in environmental microbial surveillance [15], as well as in unraveling associations between microbiomes and human diseases [16, 17]. Other applications include analyzing bacterial genome sequences to identify antimicrobial resistance genes [18, 19] and evaluating bacterial abundances to identify potential dysbiosis leading to disease [20].

The framework for amplicon 16S ribosomal RNA (16S rRNA) and metagenomic analyses has been well established in the last decade [21, 22]. Sequencing of the 16S rDNA gene subunit is a well‐established method to characterize the diversity of microbial communities and offers a cost‐effective alternative to whole genome sequencing of bacteria, archaea and some eukaryotes in one sample [23, 24]. Amplicon sequencing has become an indispensable tool for studying microbial communities, enabling researchers to analyze the composition and function of microbiomes in various environments. Popular amplicon analysis pipelines such as Mothur [25], USEARCH [26], and QIIME [21] have significantly advanced microbiome research by offering powerful functionalities for quality control, sequence processing, OTU clustering, diversity analysis, and taxonomic classification. Despite these advancements, the complexity and requirement for extensive bioinformatics knowledge remains a barrier for many researchers.

To address these challenges, last year we introduced EasyAmplicon, a user‐friendly and comprehensive amplicon analysis pipeline designed to simplify the analysis process while maintaining robust functionality. EasyAmplicon integrates multiple popular tools into a streamlined workflow, guiding users from raw data to interpretable results. By lowering the technical barrier, this pipeline aims to make advanced amplicon analysis accessible to researchers with limited bioinformatics backgrounds, thus broadening the scope and impact of microbial community studies. EasyAmplicon includes modules for efficient data processing and visualization, and it generates standard input files compatible with popular software packages such as STAMP [27], LEfSe [28], PICRUSt 1 & 2 [29, 30], BugBase [31], FAPROTAX [32], ImageGP [33], and iTOL [34]. Here, we present the entire pipeline process, detailing each step from installation and usage to the final visualization stage, specifying the input files required and the data generated as output.

## 2 APPLICATIONS OF THE METHOD

EasyAmplicon has been successfully utilized in various research projects, showcasing its versatility and practicability. Notable applications include soil microbiomes, where researchers have used EasyAmplicon to analyze the microbial diversity in agricultural soils, shedding light on the connection between soil health and nutrient cycling. In the study of gut microbiota, the pipeline has facilitated research on the gut microbiota of humans and animals, aiding in the understanding of health and disease. EasyAmplicon has also been applied to explore microbial diversity in freshwater and marine environments, contributing to ecological and environmental research. These examples highlight the broad applicability of EasyAmplicon in microbial community studies across different ecosystems.

## 3 LIMITATIONS OF THIS APPROACH

While EasyAmplicon is primarily designed for analyzing 16S rRNA amplicon sequencing data generated by second‐generation sequencing platforms (e.g., Illumina, Ion Torrent), it has limited compatibility with some third‐generation sequencing (TGS) data (e.g., PacBio). Functionality with TGS might be restricted, but future updates aim to address this limitation to ensure full compatibility with TGS data.

## 4 BIOINFORMATICS PIPELINE DESCRIPTION AND METHODS

The EasyAmplicon pipeline [35] and the graphical user interface (GUI) web server ImageGP [33] (https://www.bic.ac.cn/ImageGP/) were assembled using a combination of QIIME [4], USEARCH [26], VSEARCH [36] and in‐house scripts. These tools work together to assess sequence quality, split barcoded sequences, remove sequencing background, and identify amplicon sequence variants (ASVs) and operational taxonomic units (OTUs).

### 4.1 Computer requirement

**Figure 1:**
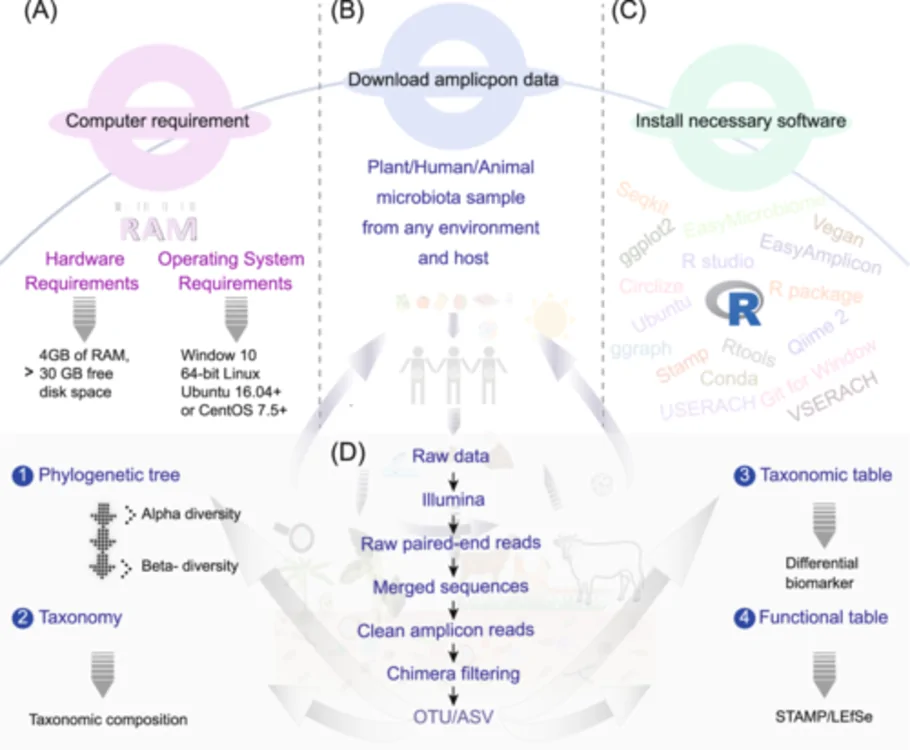
System requirements and general workflow for the EasyAmplicon pipeline. (A) To run this pipeline, system must meet the following requirements: a minimum of 4GB of RAM and 30GB or more of free disk space. Operating System: Windows 10 (64‐bit) or a compatible Linux distribution (specific Ubuntu or CentOS version). (B) The EasyAmplicon pipeline is compatible with sequencing data derived from various sources, including animal, plant, human, and soil samples. (C) Download/install software & databases such as GitBash, Rstudio, Rtools, Ubuntu, VSEARCH, USEARCH, STAMP, and QIIME2. (D) The overall workflow of the pipeline mainly constitutes three steps. (i) Dimensionality Reduction is the initial step which processes the raw sequencing reads and transforms them into a feature table. A feature table is a condensed representation of the sequencing data, summarizing the abundance of biological features (e.g., operational taxonomic units (OTUs) or amplicon sequence variants (ASVs)) found in each sample. (ii) In the analysis process, the feature table generated in the previous step serves as the foundation for various downstream analyses. Phylogenetic analysis explores the evolutionary relationships between the organisms identified in the samples. Taxonomic classification step assigns taxonomic labels (e.g., species or genus) to the OTUs or ASVs within the feature table. Functional prediction analysis leveraging the genetic data, and predicts the potential functional roles of the microbes present in the samples. Alpha diversity quantifies the species diversity within a single sample, whereas beta diversity measures the species diversity between samples. (iii) Statistics and Visualization is the final step focuses on generating high‐quality figures suitable for publication, providing visual representations of the analysis results. Statistical tests are also employed to interpret the biological significance of the findings. This pipeline generates various outputs for further biological interpretation, including phylogenetic trees depicting evolutionary relationships, taxonomic profiles detailing microbial community composition, functional profiles predicting potential metabolic functions, and alpha‐ and beta‐diversity metrics quantifying species diversity within and between samples, respectively. Importantly, our pipeline generates input files compatible with other popular microbiome analysis tools like STAMP and LEfSe, and FAPROTAX for further exploration.

- Operating system requirements: 64‐bit Linux (e.g., Ubuntu 16.04+ or CentOS 7.5+).
- Hardware requirements: At least 4 GB of RAM and 30 GB of free disk space (Figure 1A).

### 4.2 Amplicon data

Users can either utilize existing data from online repositories to initiate microbiome analysis or generate their own data by sending samples (e.g., animal, plant, human, or soil) to a sequencing company (Figure 1B).

### 4.3 Install dependency and database

Video link Tutorial: https://youtu.be/ZFrqi5P2i8M

To run the EasyAmplicon pipeline for analyzing 16S rRNA data, users must install the appropriate tools. Similar to how a carpenter needs a hammer and a painter needs a brush, we need specific tools to support our project. Install the dependency software according to the system (Windows/Mac/Linux) as mentioned in Figure 1C.

- Git for Windows 2.x.x: This tool provides an environment for window users to run shell commands, which can be used in RStudio Terminal (http://gitforwindows.org/).
- RStudio 2023.x.x: An integrated development environment for R. Choose the version that suits your system (Windows 10+ or macOS 10.14+) and download the latest version (https://posit.co/download/rstudio-desktop/).
- Editplus: A plain text editor that is small in size and has a fast startup (https://www.editplus.com/).
- R: A statistical programming language employed for data analysis and visualization (https://www.r-project.org/). Windows users may require Rtools to install R packages from source code (https://cran.rproject.org/bin/windows/Rtools/).
- STAMP v2.1.3 or later: This software is used for analyzing microbial classification and functional spectra (http://kiwi.cs.dal.ca/Software/STAMP).
- QIIME 2: A next‐generation microbiome bioinformatics platform that is extensible, free, open source, and community‐developed (https://qiime2.org).
- Conda: Software management system (https://repo.anaconda.com/miniconda).
- USEARCH v10.0.240 or later: USEARCH is a versatile tool for high‐throughput sequence analysis, offering capabilities such as clustering, chimera detection, and OTU picking. It's widely used in microbiome research for processing large datasets efficiently (http://www.drive5.com/usearch).
- VSEARCH v2.7.1+52 or later: VSEARCH is an open‐source tool for performing tasks such as sequence clustering, chimera detection, and dereplication. It's a popular alternative to USEARCH (https://github.com/torognes/vsearch).
- Users can either download the software sequentially through the official website links or directly select and download the required software from the Baidu Network Disk (https://pan.baidu.com/s/1Ikd_47HHODOqC3Rcx6eJ6Q?pwd=0315).

Note: Importantly, USEARCH and VSEARCH databases by default saved in the database directory (EasyMicrobiome).

### 4.4 Install EasyAmplicon process

To get started with the EasyAmplicon pipeline [35] for amplicon data analysis, you need to download the EasyAmplicon software and its accompanying database directory, EasyMicrobiome. We highly recommend downloading them from the official GitHub repository at https://github.com/YongxinLiu/EasyAmplicon. This ensures that users have the latest and most secure version of the software and databases for amplicon analysis. Downloading from GitHub is a straightforward process: simply access the EasyAmplicon/EasyMicrobiome repository, click the “Code” button, and choose “Download ZIP.” Save the downloaded file to your preferred location on your local disk (such as the C: or D: drive). Ensure the directory name remains unchanged after downloading; for EasyAmplicon, keep the directory name as “EasyAmplicon‐master.” For the database directory, after unzipping the file, rename the unzipped folder from “EasyMicrobiome‐master” to “EasyMicrobiome.” An alternative download option exists via Baidu Netdisk: https://pan.baidu.com/s/1Ikd_47HHODOqC3Rcx6eJ6Q?pwd=0315 (an account must be created first). Once the account is created successfully, users can download and save the EasyAmplicon process, windows packages, and dependent software all at once with one click.

Note: It is recommended to obtain the latest Baidu Netdisk link from GitHub, as the software links are frequently updated.

### 4.5 Downloading R package

The statistics and visualization may require more than 500R packages (https://cran.r-project.org/web/packages/available_packages_by_name.html#available-packages-B). Installation is time‐consuming and may depend on additional compilation tools. To simplify this process, users can directly download the necessary R packages from this link: http://www.imeta.science/db/win/4.3.zip. Alternatively, users can download R packages from Baidu Netdisk (https://pan.baidu.com/s/1Ikd_47HHODOqC3Rcx6eJ6Q?pwd=0315), located inside db/win/4.x. zip or db/mac/R4.x_mac_libraryX86_64. zip. After downloading, unzip the files and place the 4.x folder in your system at C:\Users[$UserName]\AppData\Local\R\win‐library\to complete the installation. The 4.x. zip package encompasses a comprehensive collection of frequently utilized R packages. By extracting it, users can bypass the downloading and installation processes. After launching RStudio, first go to the menu Tools, then navigate to Install Packages to view the default installation directory for R packages. Copy the compressed file 4.x. zip to the win‐library directory. Select 4.x. zip, right‐click, and choose “Extract to the current folder.” If prompted to replace files, select “Yes” for all. Restart RStudio after the replacement, and enter library (ggplot2) at the “>” prompt in the console at the lower left corner to test. If the following message appears, it means the installation was successful.

[Workspace loaded from/var/www/html/ehbio_doc/train/. RData]

> library (ggplot2)

In case of an error like “Error in library (“ggplot2”): there is no package called “ggplot2,” it means that the package was not installed successfully. This could be due to the previous extraction being unsuccessful or being placed in the wrong location. Please carefully check and repeat the above extraction and installation steps or you can use “install. packages” to install a new package, such as the following command:

install.packages("*devtools*")

if (!requireNamespace("BiocManager", quietly = TRUE))

install.packages("BiocManager")

BiocManager::install("edgeR")

library(devtools)

devtools::install_github("*microbiota*/*amplicon*")

For Mac users, extract the compressed folder from the ‘Mac’ directory to your download folder. After that, execute the following command:

cp ‐r ~/Downloads/library/*

/Library/Frameworks/R.framework/Versions/4.x/Resources/library/

Note: The EasyAmplicon pipeline might encounter errors during code execution if subfolders exist within the working directory (EasyAmplicon), the database directory (EasyMicrobiome), or the 4.3 packages folder. Nested directory structures within these locations can disrupt file path expectations and hinder the code's ability to access necessary files.

### 4.6 Extension software and default databases

Various software tools and databases required to run this pipeline are configured in the database directory—EasyMicrobiome. Users can download and use them directly or reconfigure EasyMicrobiome after obtaining the latest versions from official websites, including GitHub and Baidu Netdisk. This pipeline utilizes multiple software tools and databases, such as UNITE, RDP, SILVA, and Greengenes. These databases provide crucial information for classifying and identifying bacterial sequences.

The UNITE database (https://unite.ut.ee/) is used for internal transcribed spacer (ITS) sequencing analysis of fungi or eukaryotes [37] and is saved by default in the EasyMicrobiome/usearch directory. The Ribosomal Database Project (RDP) [38] is located by default in the EasyMicrobiome database folder. SILVA [39] and Greengenes [40] databases can be downloaded and installed for 16S microbiome analysis following the instructions provided in the QIIME 2 analysis section. Alternatively, users can download Greengenes database (https://ftp.microbio.me/greengenes_release/current/) in USEARCH‐compatible format from the official USEARCH website (http://www.drive5.com/usearch/manual/sintax_downloads.html). The SILVA database is also available for download in various formats, with instructions and download options provided on the SILVA website at https://www.arb-silva.de/download/.

### 4.7 Input data files

The input data files for this protocol include experimental design metadata and raw sequencing data. The raw sequencing data used in this paper is available in zipped FASTQ format within the “seq” folder, and a backup can be downloaded via the metadata from the Genome Sequence Archive (GSA) at https://ngdc.cncb.ac.cn/gsa/. This protocol assumes that the raw sequencing data is stored in the “seq” folder and is provided in zipped FASTQ format; however, adjustments may be necessary based on specific data storage and format requirements. If users prefer to utilize a web server, it is recommended to deposit data in GSA, as this significantly reduces download times. Alternatively, public data centers such as the National Center for Biotechnology Information and the European Bioinformatics Institute (https://www.ebi.ac.uk/) can also be employed to provide download links for online analysis.

wget ‐c ftp://download.big.ac.cn/gsa/CRA002352/CRR117575/CRR117575_f1.fq.gz ‐O seq/KO1_1.fq.gz

gunzip *.gz

### 4.8 Downloading and installing Conda on the system

The execution of EasyAmplicon script commands is conducted within the Git Bash terminal, while various analytical commands are executed within the Bash terminal of the Windows Subsystem for Linux. To use WSL, users need to perform a series of steps. First, go to Settings and type “Turn Windows features on or off” in the search bar, then enable the “Windows Subsystem for Linux” option. Next, search for your preferred Linux distribution (e.g., Ubuntu) in the Microsoft app store, select the distribution you want, and click “Get” to install it. After successful installation, users should open a terminal in the Ubuntu WSL environment and create an account. After successfully activating Ubuntu, start installing the Conda environment. For this, users need to install and activate Conda [41].

(i) To download and install Conda, open the Ubuntu and run the following commands:
wget ‐c https://repo.anaconda.com/miniconda/Miniconda3-latest-Linux-x86_64.sh

bash Miniconda3‐latest‐Linux‐x86_64.sh ‐b ‐f
~/miniconda3/condabin/conda init
(ii) Initialize Conda: Close and reopen the terminal window to apply the changes from the installer. Afterward, proceed to install QIIME 2. This method utilizes the Conda package manager for the online installation of QIIME 2. Begin by defining the desired QIIME version; here, we'll use qiime2‐amplicon‐2024.5 as an example.
n=qiime2‐amplicon‐2024.5

Next, download the list of software dependencies required for the QIIME installation:

wget ‐c https://data.qiime2.org/distro/amplicon/${n}-py39-linux-conda.yml

If the primary source fails, run the following command as an alternative:

wget ‐c http://www.imeta.science/db/conda/${n}-py39-linux-conda.yml

Subsequently, to create a new Conda environment named after the chosen QIIME version and install the dependencies listed in the downloaded YAML file, run this code:

conda env create ‐n ${n} ‐‐file ${n}‐py39‐linux‐conda.yml

This step (approximately 1.2 GB) is optional and allows users to package the installed environment for distribution on computer servers.

conda pack ‐n ${n} ‐o ${n}.tar.gz

Finally, activate and initialize the QIIME 2 environment:

conda activate ${n}

Note: If users encounter difficulties in this step, they can open a web browser and visit the Miniconda website (https://docs.conda.io/en/latest/miniconda.html), which provides comprehensive installation instructions. Locate the section corresponding to your operating system (e.g., Windows, macOS, and Linux). Within that section, search downloads links for the most recent Miniconda3 installer. The filename typically incorporates “Miniconda3‐latest” and user system type (e.g., “x86_64”). Replace Linux‐x86_64 with your specific type (e.g., osx‐x86_64 for macOS) if necessary. Users can find a list of available options depending on system configuration on the Miniconda download page. For Ubuntu users on Windows 10 plus, please download the Linux 64‐bit installer.

### 4.9 R setup

- Using Windows 10+ as example.
- Select the Tools menu —— Global Options.
- Set the default working directory.
- General —— Default working directory —— choose C: or D:/Easymplicon or amplicon (any name).
- Code —— Saving —— Default text encoding —— chooseUTF‐8.
- Mirror selection: speed up installation package download (optional).
- Packages —— CRAN mirror —— choose Beijing, otherwise Hefei/Guangzhou/Lanzhou/Shanghai optional (choose CRAN mirror nearest to your location).
- Switch the setting and set terminal as Git Bash by following this route; Open RStudio —— Tools —— Global Options —— Terminal —— New terminals —— Git Bash —— OK). Close the file, exit the program, then reopen RStudio to restore normalcy.
- Set working directory in Rstudio; click on Session —— Set working directory —— choose directory and open your working folder.
- File —— Open file —— EasyAmplicon master folder —— pipeline. sh (windows/linux) or pipeline_mac. sh (for mac users).

## 5 SUMMARY

EasyAmplicon offers flexibility in data input, accepting various formats like paired‐end or single‐end sequencing reads (FASTQ), clean amplicons (FASTA). The pipeline typically starts by merging paired‐end reads, removing primers and barcodes, and filtering low‐quality reads to obtain clean amplicons, primarily using VSEARCH or USEARCH software. Clean amplicons can then be clustered into OTUs based on 97% similarity, denoised into ASVs using a de novo approach, or directly mapped to a reference database like Greengenes for closed‐reference OTU tables. Following OTU/ASV generation, EasyAmplicon builds a feature table summarizing the abundance of these biological features in each sample. The feature table, combined with a phylogenetic tree built from representative sequences, drives downstream analyses such as alpha and beta diversity assessments, taxonomic classification, functional prediction of microbial activity, and biomarker discovery to highlight distinguishing features between sample groups (Figure 1D). For better understanding, users can refer to the video tutorial links provided in each section.

## 6 PROCEDURE

Video link tutorial: https://www.youtube.com/watch?v=9ORamd84hUc.

### 6.1 Quick start

This section details the initial steps for setting up the environment to run the EasyAmplicon pipeline. Begin by setting the working directory (wd) to amplicon/EasyAmplicon and the software database directory (db) to EasyMicrobiome. Avoid using the files downloaded directly from GitHub as your working directory. Instead, create a new folder named amplicon/EasyAmplicon and designate it as your working directory. Follow the instructions provided in the pipeline scripts (pipeline. sh for Chinese, pipeline_en. sh for English, and pipeline_mac. sh for Mac users) to correctly set the paths for $wd and $db. Execute the code line by line or section by section in RStudio to complete the analysis process. Each time you open RStudio, ensure to run the following four lines of code to establish the working and database directories.

wd=/c/EasyAmplicon

db=/c/EasyMicrobiome

PATH=$PATH:${db}/win

cd ${wd}

Note: Optionally, users can substitute $wd and db with the installation path of EasyAmplicon and EasyMicrobiome, located on either drive C or drive D. For detailed guidance on setting environment variables, users should refer to the pipeline script.

### 6.2 Dimensionally reduction (from raw data to tables)

#### 6.2.1 Preparing files

**Figure 2:**
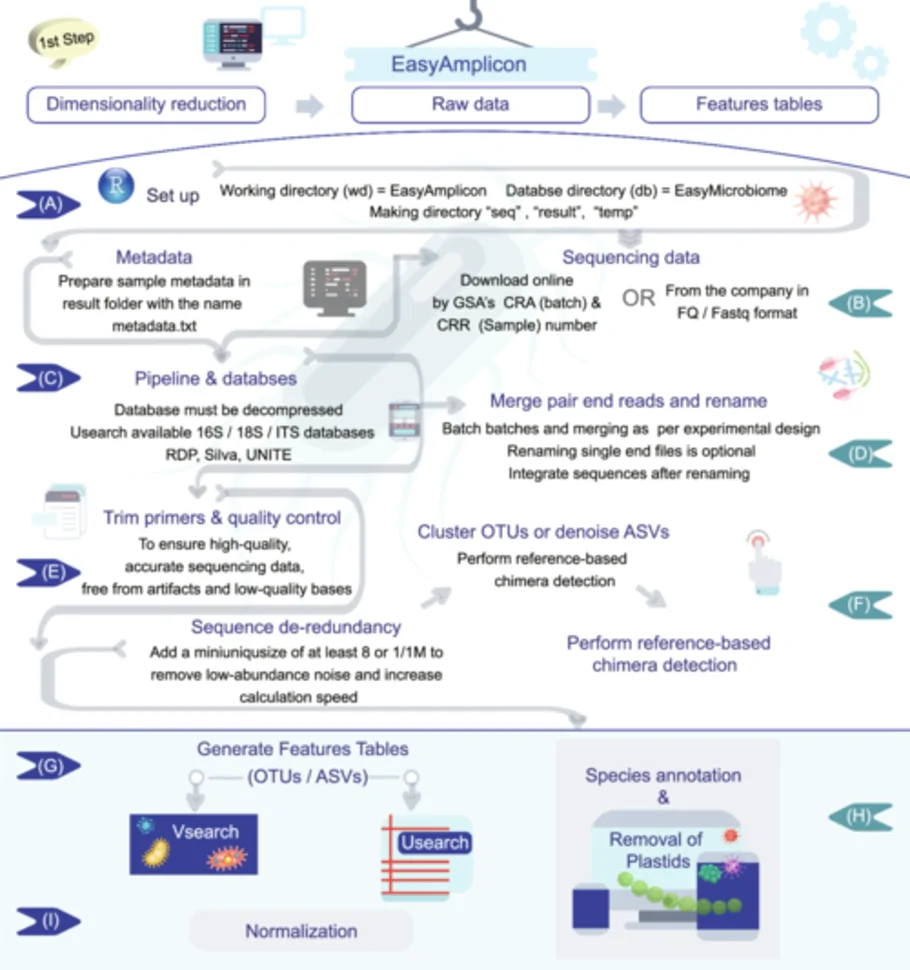
In the Pipeline, the first step is the dimensionality reduction, which processes raw data into feature tables. (A) Initially, set up the working directory (wd) for EasyAmplicon and the database directory (db) for EasyMicrobiome. (B) Users must have “metadata.txt” saved in the “result” directory and sequencing data FASTQ files saved in the “seq” directory, typically ending with.fq.gz, with one pair of files per sample. Decompress.gz files using gunzip if required (VSEARCH can often read them directly). (C) EasyAmplicon utilizes pre‐built reference databases for sequence analysis. These databases include RDP, SILVA, and UNITE (by default, saved inside the EasyMicrobiome folder). The database must be decompressed the first time it is used, and you can skip this section later. (D) Then, reads are merged and renamed according to the experimental design. (E) Subsequently, this step involves rigorous quality control measures, filtering out low‐quality reads based on various criteria, such as sequencing errors and base quality scores. (F) Sequence dereplication is performed to remove duplicate sequences and clustering or denoising is conducted to group similar sequences into operational taxonomic units or amplicon sequence variants, respectively. Additionally, reference‐based chimera detection helps identify and eliminate artifacts. (G) A feature table is generated using software tools like USEARCH and VSEARCH, containing abundance information of microbial features across samples. (H) Taxonomic annotation is applied based on reference databases like RDP, SILVA, or UNITE, while nontarget DNA sequences (plastids) are excluded. Subsequently, the tables are filtered to remove low‐quality sequences. (I) This is followed by normalization to ensure standardized comparison and analysis. This comprehensive pipeline enables efficient processing and analysis of amplicon sequencing data to characterize microbial communities accurately.

To run the pipeline, two main data files are required: sequencing data and metadata file. Sequencing data (*.fq.gz) includes paired FASTQ files stored in the seq/directory, usually compressed in.gz format to save space. Metadata (metadata.txt) provides descriptive information such as grouping, time, location, etc. corresponding to each sample number. Open pipeline.sh, which is by default located in the EasyAmplicon master folder downloaded from Github or Baidu. Then create subdirectories within working directory for sequencing data (“seq”), results (“result”), and temporary file (“temp”) by running the following code (Figure 2A).

mkdir ‐p seq result temp

Note: It is recommended to save sequencing data inside the seq directory and metadata raw text inside the result directory.

##### 6.2.1.1 Metadata/experimental design

This section guides users in checking and preparing the sample metadata file for downstream analysis.

(1)
**Verify metadata structure**
  This step involves checking the structure and formatting of the metadata file using “csvtk” package (Figure 2B), which determines the number of rows (samples, excluding the header) and columns in the table. Run the following code in RStudio terminal csvtk ‐t stat result/metadata_raw.txt. This ensures the metadata file has the necessary structure for the analysis.
  ▲Troubleshooting: If users encounter the error “CSVTK not found” when running the code, it means the csvtk command‐line tool is not available on your system. By default, csvtk saved in the EasyMicrobiome/win folder within the database directory. Ensure that there is no nested EasyMicrobiome subfolder within the primary folder, as this may lead to complications in assessment and potentially hinder code execution.
(2)
**Review files header and formatting**
  To view file header, use the cat command provided below. The head command is used to display the file header, and the “‐n3” option limits the output to the first 3 lines of the metadata file, allowing you to verify the presence of column headers and their naming conventions.
cat ‐A result/metadata_raw.txt | head ‐n3
  Note: If you are a windows user, you may notice a “^M” symbol at the end of the data, which represents the Windows‐specific carriage return character. This will need to be formatted in the next step.
(3)
**Formatting metadata file (Windows‐Specific)**
  This step addresses a potential formatting issue specific to Windows systems. The “sed” command is used to remove carriage return characters (\r) that may be present in the metadata file created on a windows system. Although these characters are not typically visible when viewing the file in text editors but can cause issues during analysis. Run the following code, replacing result/metadata_raw.txt to the result/metadata.txt, which should be a tab‐delimited text file containing sample information.
sed 's/\r//' result/metadata_raw.txt > result/metadata.txt

Next, run the code given below, which uses the cat command again to view the first few lines of the cleaned file and confirm the removal of carriage return characters.

cat ‐A result/metadata.txt | head ‐n3

By following these steps, metadata file is properly formatted and ready to use for downstream analysis.

##### 6.2.1.2 Sequencing data

This section covers two approaches for handling sequencing data required for analysis with EasyAmplicon.

(1) **Downloading from public sources**
  If sequencing data is not readily available, this pipeline allows users to download it from public repositories like the GenBank Sequence Read Archive (SRA). Here's an example using the wget command to download a specific file:
wget ‐c ftp://download.big.ac.cn/gsa/CRA002352/CRR117575/CRR117575_f1.fq.gz ‐O seq/KO1_1.fq.gz.
  Additionally, a bulk download and renaming process is facilitated based on experimental design numbers using the “awk” command. The command given below is used to explore metadata file and constructs download URLs for each sample, ensuring efficient downloading and organization of sequencing data.
awk '{system("wget ‐c ftp://download.big.ac.cn/gsa/"$5"/"$6"/"$6"_f1.fq.gz ‐O seq/"$1"_1.fq.gz")}'\
<(tail ‐n+2 result/metadata.txt)
awk '{system("wget ‐c ftp://download.big.ac.cn/gsa/"$5"/"$6"/"$6"_r2.fq.gz ‐O seq/"$1"_2.fq.gz")}'\
<(tail ‐n+2 result/metadata.txt)
  Note: Public sequencing data repositories offer a wide variety of datasets. Consult the specific repository documentation for download instructions and data formats.
(2) **Using existing sequencing data**
  This step describes how to download and prepare sequencing data for analysis in RStudio. If the sequencing data has already been generated and stored elsewhere, ensure that the paired‐end FASTQ files, compressed with.gz extension (e.g., KO1_1.fq.gz) KO1_1. fq.gz) are placed in the seq directory within working directory EasyAmplicon. The script emphasizes the importance of ensuring file names correspond to sample IDs in metadata file for accurate analysis (Figure 2B). Execute the following code:
ls ‐sh seq/
zless seq/KO1_1.fq.gz | head ‐n4

To view 1‐60 characters per line, run this code:

zless seq/KO1_1.fq | head | cut ‐c 1‐60

The provided commands like “ls ‐sh seq” and “zless,” are helpful for managing and viewing compressed files in the seq directory. To determine basic statistics about the sequencing data, such as the number of reads and their lengths, run the following code, which utilizes seqkit stat.

seqkit stat seq/KO1_1.fq.gz

seqkit stat seq/*.fq.gz > result/seqkit.txt

head result/seqkit.txt

##### 6.2.1.3 Pipeline & databases

This section of the protocol focuses on the reference databases used for downstream analyses in EasyAmplicon pipeline. Their location depends on how you downloaded them. If you downloaded the EasyAmplicon databases through Baidu Netdisk, they are likely stored in a directory named “db.” Conversely, if you downloaded them from GitHub as part of EasyMicrobiome, you can find them in the win folder. The “win” folder typically contains commonly used reference databases; however, some databases, like EzBioCloud, might not be included. If you require a specific database that is not present, you can download it from Baidu Netdisk (if available) or from a trusted online repository. Once downloaded, place the database file(s) in the EasyMicrobiome “win” folder for future use. Note that some reference databases might be initially compressed in the “.gz” format. If this is your first time downloading them, you'll need to decompress them using the “gunzip” command before proceeding. Once decompressed, you can skip this step in the future. The following code demonstrates how to decompress representative 16S/18S/ITS RDP, SILVA, and UNITES databases (Figure 2C).

gunzip ‐c ${db}/usearch/rdp_16s_v18.fa.gz > ${db}/usearch/rdp_16s_v18.fa

seqkit stat ${db}/usearch/rdp_16s_v18.fa

seqkit stat ${db}/usearch/unite.fa

The Greengene database is used for feature annotations and the default decompression will delete the original file, ‘‐c’ specifies the output to the screen, ‘>’ write a new file and rename it.

gunzip ‐c ${db}/gg/97_otus.fasta.gz > ${db}/gg/97_otus.fa

seqkit stat ${db}/gg/97_otus.fa

#### 6.2.2 Reads merge and rename

This section addresses merging and potentially renaming paired‐end sequencing data files using VSEARCH software. Merging combines the forward and reverse reads for each sample into a single file, while renaming ensures consistent naming conventions for downstream analysis (Figure 2D).

##### 6.2.2.1 Merging and renaming process

This pipeline demonstrates two main methods for merging and renaming.

**Method 1** (Sequential processing with a “for” Loop): This method is suitable for all systems and iterates through your samples listed in the metadata file, merging and renaming each pair of reads accordingly.

time for i in 'tail ‐n+2 result/metadata.txt|cut ‐f1';do

vsearch ‐‐fastq_mergepairs seq/${i}_1.fq.gz ‐‐reverse seq/${i}_2.fq.gz\

‐‐fastqout temp/${i}.merged.fq ‐‐relabel ${i}.

done &

**Method 2** (Parallel processing with “rush”): This method leverages the “rush” command to perform parallel processing, potentially improving speed on systems with multiple cores. Ensure that your system supports “rush” before using this method, as some computers do not support it and may encounter scheduling failure during execution. If that's the case, please use the “for” loop section instead).

time tail ‐n+2 result/metadata.txt | cut ‐f 1 |\

rush ‐j 3 "vsearch ‐‐fastq_mergepairs seq/{}_1.fq.gz ‐‐reverse seq/{}_2.fq.gz\

‐‐fastqout temp/{}.merged.fq ‐‐relabel {}."

To check the sample names in the first 10 lines of the last file, run this code:

head temp/'tail ‐n+2 result/metadata.txt | cut ‐f 1 | tail ‐n1'.merged.fq | grep ^@

Note: Regardless of the method chosen, ensure the script or loop execution completes before proceeding to the next step. Incomplete processing can lead to inconsistencies in data.

##### 6.2.2.2 Integrating sequences after renaming

The final step combines all merged and renamed files into a single file named all.fq within the temp directory by running the code “cat temp/*.merged.fq > temp/all.fq.” To check the size of the merged file, use this command “ls ‐lsh temp/all.fq.” Ensure that sample names appear correctly before the dot (.) in the sequence headers by using the code “head ‐n 6 temp/all.fq | cut ‐c1‐60.” This helps avoid memory issues during subsequent analyses.

#### 6.2.3 Trim primers and perform quality control

This section describes trimming primer sequences and performing quality control on the merged and renamed reads (Figure 2E). Here, the primer lengths are assumed to be 29 bp on the left and 18 bp on the right. Be sure to adjust these values based on your actual primer design.

time vsearch ‐‐fastx_filter temp/all.fq ‐‐fastq_stripleft 29 ‐‐fastq_stripright 18 ‐‐fastq_maxee_rate 0.01 ‐‐fastaout temp/filtered.fa

#### 6.2.4 Remove redundancy and select OTU/ASV

This section covers two key steps: de‐redundancy and OTU/ASV selection (Figure 2F). The de‐redundancy process removes identical sequences, retaining only a single copy along with its abundance information. This reduces file size and speed up further analysis. The OTU/ASV selection step clusters similar sequences into operational taxonomic units or amplicon sequence variants. OTUs represent clusters of sequences with high similarity (typically 97% or greater), while ASVs aim for single‐nucleotide accuracy.

##### 6.2.4.1 De‐redundancy

Run the command “vsearch ‐‐derep_fulllength temp/filtered.fa ‐‐minuniquesize 10 ‐‐sizeout ‐‐relabel Uni_ ‐‐output temp/uniques.fa.” The provided code demonstrates how to use VSEARCH to dereplicate sequences. The “‐‐derep_fulllength” option identifies and removes identical sequences. “‐‐minuniquesize” sets a minimum abundance threshold to exclude very rare sequences (considered noise). A value of 10 is commonly used. “‐‐sizeout” outputs a table containing sequence abundance information. “‐‐relabel Uni”_ adds a prefix to the sequence names for better organization. The command generates two files: (i) temp/uniques. fa (this file contains the de‐redundant sequences), (ii) temp/uniques. fa. sizeout (this file contains the abundance information for each sequence.)

Note: Consider adjusting the “‐‐minuniquesize” value based on data and analysis requirements. A higher threshold removes more low‐abundance sequences.

##### 6.2.4.2 Cluster OTUs or denoise ASVs

This section discusses two main approaches for grouping similar sequences comprising OTUs clustering [42, 43] and ASV denoising [44, 45]. Both methods within VSEARCH include built‐in de novo chimera removal to eliminate artificial sequences formed during PCR amplification.

**Method 1** (97% OTUs Clustering): This method is suitable for large datasets, when ASVs patterns are unclear, or when requested by a reviewer.

usearch ‐cluster_otus temp/uniques.fa ‐minsize 10\

‐otus temp/otus.fa\

‐relabel OUT_

**Method 2** (ASV denoising): To identify exact biological sequences while removing sequencing errors and to obtain precise sequence information, execute this code:

usearch ‐unoise3 temp/uniques.fa ‐minsize 10 ‐zotus temp/zotus.fa

To modify sequence names: change ‘Zotu’ to ‘ASV’ for easier identification, run the given code:

sed 's/Zotu/ASV_/g' temp/zotus.fa > temp/otus.fa

head ‐n 2 temp/otus.fa

Note: Processing time for ASV denoising can vary depending on dataset size. Use OTU clustering for large datasets or when exact sequence information is not essential. Use ASV denoising for studies that require high‐precision sequence data (e.g., microbial community diversity analysis).

##### 6.2.4.3 Reference‐based chimera detection

Generally chimera removal is not recommended, as it can lead to false negatives due to the absence of abundance information in the reference database. In contrast, de novo chimera detection requires the parent abundance to be at least 16 times that of the chimera to prevent false negatives. Since known sequences will not be removed, choosing a larger reference database is more appropriate to minimize the false negative rate.

mkdir ‐p result/raw

**Method 1:** Using VSEARCH in combination with RDP for chimera removal (fast but prone to false negatives), download and extract the SILVA database (replacing rdp_16s_v18. fa with silva_16s_v123. fa). This process is very slow but theoretically provides better results. Use the following command:

vsearch ‐‐uchime_ref temp/otus.fa\

‐db ${db}/usearch/rdp_16s_v18.fa\

‐‐nonchimeras result/raw/otus.fa

If you're using Windows, VSEARCH results may have added Windows line endings (^M), which you need to remove them. However, if you're using a Mac, you don't need to execute this command:

sed ‐i 's/\r//g' result/raw/otus.fa

**Method 2:** This method provides an alternative approach where chimera removal is not performed. This can be useful if you've already addressed chimeras in a previous step or for specific downstream analyses requiring the unfiltered OTUs.

cp ‐f temp/otus.fa result/raw/otus.fa

#### 6.2.5 Feature table construction and filtering

This section covers generating a feature table, which summarizes the abundance of features (OTUs or ASVs) found in each sample. It also details optional steps for species annotation and filtering based on taxonomic classification (Figure 2G).

##### 6.2.5.1 Generate feature table as per sample size

The choice of method for generating the feature table depends on dataset size. There are two methods to generate feature table.

**Method 1:** This method uses USEARCH to generate feature table. Its fast for small samples (<30) but becomes inefficient for larger datasets.

time usearch ‐otutab temp/filtered.fa

‐otus result/raw/otus.fa

‐threads 4

‐otutabout result/raw/otutab.txt

**Method 2**: This method utilizes VSEARCH to efficiently generate a feature table summarizing the read counts for each OTU and particularly well‐suited for handling large datasets.

time vsearch ‐‐usearch_global temp/filtered.fa

‐‐db result/raw/otus.fa

‐‐id 0.97 ‐‐threads 4

‐‐otutabout result/raw/otutab.txt

The processing time for feature table generation can vary depending on the chosen method, data set size, and available computational resources. VSEARCH offers multi‐threading capabilities to improve processing speed. The output feature table (result/raw/otutab.txt) will contain abundance information for each feature (OTU/ASV) in each sample.

##### 6.2.5.2 Species annotation – removal of plastids and nonbacterial/archaea (Optional)

This section covers steps for annotating features with species information and filtering the data based on taxonomic classification (Figure 2H). For species annotation, use a reference database like RDP (faster) or SILVA (more comprehensive) to assign potential species classifications to features.

vsearch ‐‐sintax result/raw/otus.fa\

‐‐db ${db}/usearch/rdp_16s_v18.fa\

‐‐sintax_cutoff 0.1\

‐‐tabbedout result/raw/otus.sintax

head result/raw/otus.sintax | cat ‐A

sed ‐i 's/\r//' result/raw/otus.sintax

**Method 1** (Filtering based on taxonomy): This method explains how to filter data based on taxonomic annotations. R script is provided to exclude nonbacterial/archaeal sequences (e.g., plastids and mitochondria) from the feature table (result/raw/otutab.txt) and species annotation file (result/raw/otus.sintax). For fungal ITS data specifically, use the “otutab_filter_nonFungi.R.”

wc ‐l result/raw/otutab.txt

Rscript ${db}/script/otutab_filter_nonBac.R

‐‐input result/raw/otutab.txt\

‐‐taxonomy result/raw/otus.sintax\

‐‐output result/otutab.txt\

‐‐stat result/raw/otutab_nonBac.stat\

‐‐discard result/raw/otus.sintax.discard

To count the number of filtered feature table rows. Run this code:

wc ‐l result/otutab.txt

To filter the sequence corresponding to the feature table, run this code:

cut ‐f 1 result/otutab.txt | tail ‐n+2 > result/otutab.id

usearch ‐fastx_getseqs result/raw/otus.fa\

‐labels result/otutab.id ‐fastaout result/otus.fa

To filter feature tables corresponding to sequence annotations, run the following code:

awk 'NR==FNR{a[$1]=$0}NR>FNR{print a[$1]}'

result/raw/otus.sintax result/otutab.id

> result/otus.sintax

**Method 2:** This method provides an option to skip additional filtering of the generated feature table (e.g., abundance thresholds and taxonomic filtering) if the initial screening in a previous step was sufficient.

cp result/raw/otu* result/

To get the OTUs summary table (optional), run this code:

usearch ‐otutab_stats result/otutab.txt\

‐output result/otutab.stat

cat result/otutab.stat

##### 6.2.5.3 Sample equalization

This step addresses normalizing the read counts across samples to account for potential differences in sequencing depth (Figure 2I). The provided R script utilizes the vegan package to perform sample equalization. The output will be “otutab_rare.txt” and “vegan.txt,” which can be used to plot alpha diversity in downstream analyses.

mkdir ‐p result/alpha

Rscript ${db}/script/otutab_rare.R ‐‐input result/otutab.txt\

‐‐depth 10000 ‐‐seed 1\

‐‐normalize result/otutab_rare.txt\

‐‐output result/alpha/vegan.txt

usearch ‐otutab_stats result/otutab_rare.txt\

‐output result/otutab_rare.stat

cat result/otutab_rare.stat

Note: As this pipeline is tailored for end‐to‐end analysis, the files generated in this section will be used to generate alpha diversity plots in the subsequent analysis. A detailed overview of all preceding steps is illustrated in Figure 2.

### 6.3 Analysis (from big tables to small tables)

Video link tutorial: https://youtu.be/R7wj3D9B6xU

**Figure 3:**
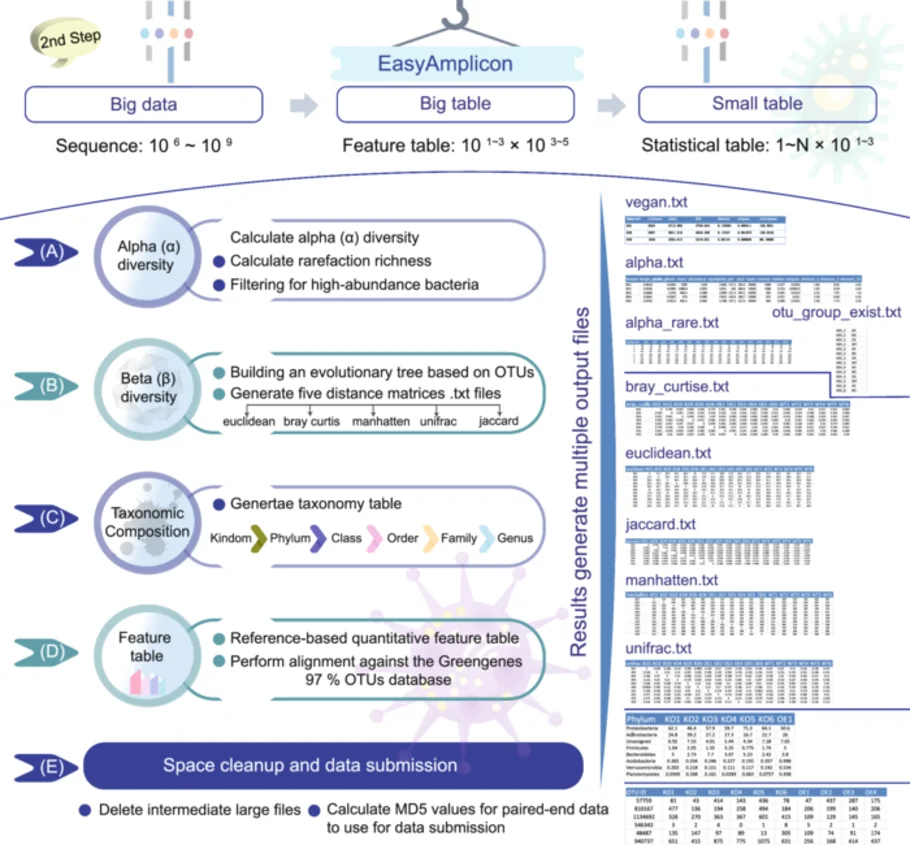
EasyAmplicon pipeline steps for taxonomy diversity analysis. (A) Alpha diversity involves analyzing species diversity within a sample to summarize microbial diversity. The outputs include alpha.txt, alpha_rare.txt, and OTU_group_exist files. (B) Beta diversity involves analyzing species diversity between samples to summarize microbial diversity across different samples. The outputs include text files of distance matrices: bray_curtis.txt, euclidean.txt, jaccard.txt, manhattan.txt, and unifrac.txt. These files contain distance matrices that measure the dissimilarity between samples in terms of their microbial communities. (C) Taxonomic composition: This step classifies microbes into taxonomic groups, including phylum, class, order, family, and genus. (D) Feature table: This table contains abundance and taxonomic information, with alignment performed against the Greengenes database (97% OTUs). (E) Space cleanup and data submission is the final step, which involves removing temporary files and preparing data for submission to public repositories, including calculating MD5 values for paired‐end data. OTU, operational taxonomic unit.

Feature tables are a critical output obtained during the data processing step in microbiome analysis. They condense the sequencing data by summarizing how many reads (sequences) from each sample map to specific OTUs. These tables are crucial for calculating diversity within samples (α‐diversity) and between samples (β‐diversity) [46, 47]. Additionally, incorporating taxonomic information allows researchers to explore specific groups of microbes (e.g., genus and family) and potentially identify biomarkers linked to certain conditions (Figure 3). Feature tables also enable further analysis like differential abundance testing, which identifies microbes significantly associated with specific conditions or states.

#### 6.3.1 Alpha (α) diversity analysis

##### 6.3.1.1 Calculation of alpha diversity indices

This step focuses on calculating α‐diversity, a metric that quantifies species richness and evenness within a sample (Figure 3A). USEARCH, a versatile tool included in this pipeline which can compute 14 different alpha diversity indices. Refer to the USEARCH manual (http://www.drive5.com/usearch/manual/alpha_metrics.html) for detailed descriptions of these indices, and run the given code in R which utilize “otutab_rare.txt” containing the data for alpha diversity calculation and generate output file “alpha. txt.”

usearch ‐alpha_div result/otutab_rare.txt ‐output result/alpha/alpha.txt

##### 6.3.1.2 Rarefaction richness analysis

The pipeline utilizes a dilution curve method, which involves subsampling sequencing data at various percentages (often ranging from 1% to 100%). To assess the diversity of microbial communities while compensating for differences in sequencing depth across samples, perform a rarefaction analysis. This analysis estimates the expected number of OTUs that would be identified at a specific sequencing depth (number of reads sequenced per sample). Execute the following code which utilizes USEARCH to generate a file named “alpha_rare.txt” containing rarefaction richness data:

usearch ‐alpha_div_rare result/otutab_rare.txt ‐output result/alpha/alpha_rare.txt

‐method without_replacement

head ‐n2 result/alpha/alpha_rare.txt

sed ‐i "s/‐/\t0.0/g" result/alpha/alpha_rare.txt

At each subsampling depth, the “without_replacement” option ensures each OTU has an equal probability of being included, mitigating biases from uneven sequencing depth.

##### 6.3.1.3 High abundance bacteria filtering (Optional)

This step allows users to filter out highly abundant bacteria from sample data. This can be useful for focusing downstream analyses on less abundant taxa that might be masked by dominant species. Group column names are adjusted based on the experimental design outlined in the metadata.txt file. The feature table (result/otutab.txt) and the metadata file (metadata.txt) are used as input. The output is a modified feature table containing group‐wise means. Use the code “Rscript ${db}/script/otu_mean.R ‐h,” In which “‐h” option displays the script's help, providing explanations for the parameters. The parameter “scale” determines whether to perform scaling, “zoom” normalizes the total sum, “all” outputs mean for all samples, and ‘type’ specifies the calculation type as “mean” or “sum.”

Rscript ${db}/script/otu_mean.R ‐‐input result/otutab.txt

‐‐metadata result/metadata.txt\

‐‐group Group ‐‐thre 0\

‐‐scale TRUE ‐‐zoom 100 ‐‐all TRUE ‐‐type mean\

‐‐output result/otutab_mean.txt

The output includes both the overall mean and the mean values for each group.

head ‐n3 result/otutab_mean.txt

By filtering with an average abundance threshold of > 0.1%, which can be selected as 0.5% or 0.05%, will obtain the OTU combinations for each group.

awk 'BEGIN{OFS=FS=“\t"}{if(FNR==1) {for(i=3;i<=NF;i++) a[i]=$i; print "OTU","Group";}

else {for(i=3;i<=NF;i++) if($i>0.1) print $1, a[i];}}'

result/otutab_mean.txt > result/alpha/otu_group_exist.txt

head result/alpha/otu_group_exist.txt

cut ‐f 2 result/alpha/otu_group_exist.txt | sort | uniq ‐c

Note: Alternatively, explore this link http://ehbio.com/test/venn/ for online analysis of OTU/ASV counts across groups at various abundance levels. This tool can generate informative Venn or network diagrams to visualize shared and unique components within each group.

#### 6.3.2 Beta (β) diversity

β‐diversity is an ecological concept that specifically refers to the difference in species composition between different groups or ecological niches [48, 49]. In the field of metagenomics, scatterplots are commonly used to display diversity between sample groups. Common analysis methods include principal component analysis (PCA), principal coordinate analysis (PCoA/MDS), and constrained/canonical PCoA (CPCoA/CCA/RDA) [50, 51, 52]. The calculation of PCoA depends on a distance matrix, which quantifies the dissimilarity between pairs of samples based on their species composition. A commonly used distance metric is the Unifrac distance, which accounts for both the presence or absence of species and their evolutionary relationships, as represented by a phylogenetic tree [53] (Figure 3B). Execute mkdir ‐p result/beta/ command to create a directory named “beta” within the result folder to store the beta diversity output files. To construct a phylogenetic tree, run usearch ‐cluster_agg result/otus.fa ‐treeout result/otus.tree command which utilizes USEARCH software to construct a phylogenetic tree based on the OTU sequences stored in the “result/otus.fa” file. The generated tree will be saved as “result/otus.tree.” The next command uses USEARCH to generate five different distance matrices based on the OTU table (“result/otutab_rare.txt”) and the constructed phylogenetic tree (”result/otus.tree”). The distance matrices will be saved with the prefix “result/beta/” followed by the specific distance metric name (e.g., ‘“bray_curtis,” “euclidean,” etc.).

usearch ‐beta_div result/otutab_rare.txt ‐tree result/otus.tree\

‐filename_prefix result/beta/

##### 6.3.2.1 Compilation of species annotation classification

To compile species annotation classifications, reformat the OTU corresponding species annotations into a two‐column structure, run the code given below. This process entails removing confidence values from SINTAX, retaining species annotations, replacing “:” with “_,” and removing any quotation marks.

cut ‐f 1,4 result/otus.sintax\

|sed 's/\td/\tk/;s/:/__/g;s/,/;/g;s/"//g'

> result/taxonomy2.txt

head ‐n3 result/taxonomy2.txt

Run the following code to generate a species table where blanks in the OTU/ASV are filled with “Unassigned.”

awk 'BEGIN{OFS=FS=“\t"}{delete a; a["k"]="Unassigned";a["p"]="Unassigned";a["c"]="Unassigned";a["o"]="Unassigned";a["f"]="Unassigned";a["g"]="Unassigned";a["s"]="Unassigned";

split($2,x,";");for(i in x){split(x[i],b,"__");a[b[1]]=b[2];}

print $1,a["k"],a["p"],a["c"],a["o"],a["f"],a["g"],a["s"];}'

result/taxonomy2.txt > temp/otus.tax

sed 's/;/\t/g;s/.__//g;' temp/otus.tax|cut ‐f 1‐8 |\

sed '1s/^/OTUID\tKingdom\tPhylum\tClass\tOrder\tFamily\tGenus\tSpecies\n/'\

> result/taxonomy.txt

head ‐n3 result/taxonomy.txt

Next, execute the following commands to count the number of taxa at different ranks: phylum, class, order, family, and genus. Use the rank parameters p, c, o, f, and g, respectively (Figure 3C). This will help categorize and quantify the microbial community across various taxonomic levels, providing a detailed view of its composition.

mkdir ‐p result/tax

for i in p c o f g;do

usearch ‐sintax_summary result/otus.sintax\

‐otutabin result/otutab_rare.txt ‐rank ${i}\

‐output result/tax/sum_${i}.txt

done

sed ‐i 's/(//g;s/)//g;s/\"//g;s/\#//g;s/\/Chloroplast//g' result/tax/sum_*.txt

To list all files, run this code:

wc ‐l result/tax/sum_*.txt

head ‐n3 result/tax/sum_g.txt

Note: The output file generated here “sum_p.txt” will be used in the coming step to construct stacked histogram, and c sum_c.txt for chord diagram.

#### 6.3.3 Functional prediction alignment

To generate a reference‐based quantitative feature table, align the sequences against the Greengenes 97% OTUs database for use in PICRUSt/Bugbase functional prediction (Figure 3D). The first step is to create a directory to store the alignment results: mkdir ‐p result/gg/. Multiple methods can be used for performing this step depending on size.

**Method 1:** By using USEARCH alignment, this method offers faster processing speed. However, consider file size limitations, especially with large datasets. The example demonstrates alignment with 4 threads, resulting in an 80.0% alignment rate. Processing times and memory usage vary depending on hardware specifications.

usearch ‐otutab temp/filtered.fa ‐otus ${db}/gg/97_otus.fa ‐otutabout result/gg/otutab.txt ‐threads 4

**Method 2:** VSEARCH alignment offers higher accuracy but requires more processing time. It leverages parallelization for improved performance, especially with 24‐96 threads. The commented example demonstrates vsearch alignment with 12 threads, achieving an 81.04% alignment rate.

vsearch ‐‐usearch_global temp/filtered.fa ‐‐db ${db}/gg/97_otus.fa ‐‐otutabout result/gg/otutab.txt ‐‐id 0.97 ‐‐threads 12

To find the statistics, run this code:

usearch ‐otutab_stats result/gg/otutab.txt ‐output result/gg/otutab.stat

cat result/gg/otutab.stat

#### 6.3.4 Clean‐up and data submission

This step covers post‐analysis clean‐up and data preparation for submission (Figure 3E). First, remove temporary files to free up storage space by running “rm ‐rf temp/*.fq” command. Then, use double‐ended statistical md5 values for data submission:

cd seq

md5sum *_1.fq.gz > md5sum1.txt

md5sum *_2.fq.gz > md5sum2.txt

paste md5sum1.txt md5sum2.txt | awk '{print $2"\t"$1"\t"$4"\t"$3}'

| sed 's/*//g' >../result/md5sum.txt

rm md5sum*

cd..

cat result/md5sum.txt

### 6.4 Statistics and visualization of plots

Video tutorial link: https://youtu.be/n13M8p_IrXk

In continuation with the pipeline, this section “diversity plot under R (command line mode)” demonstrates the scripts to generate plots for alpha diversity, beta diversity, and species composition taxonomy.

#### 6.4.1 Alpha diversity plotting

**Figure 4:**
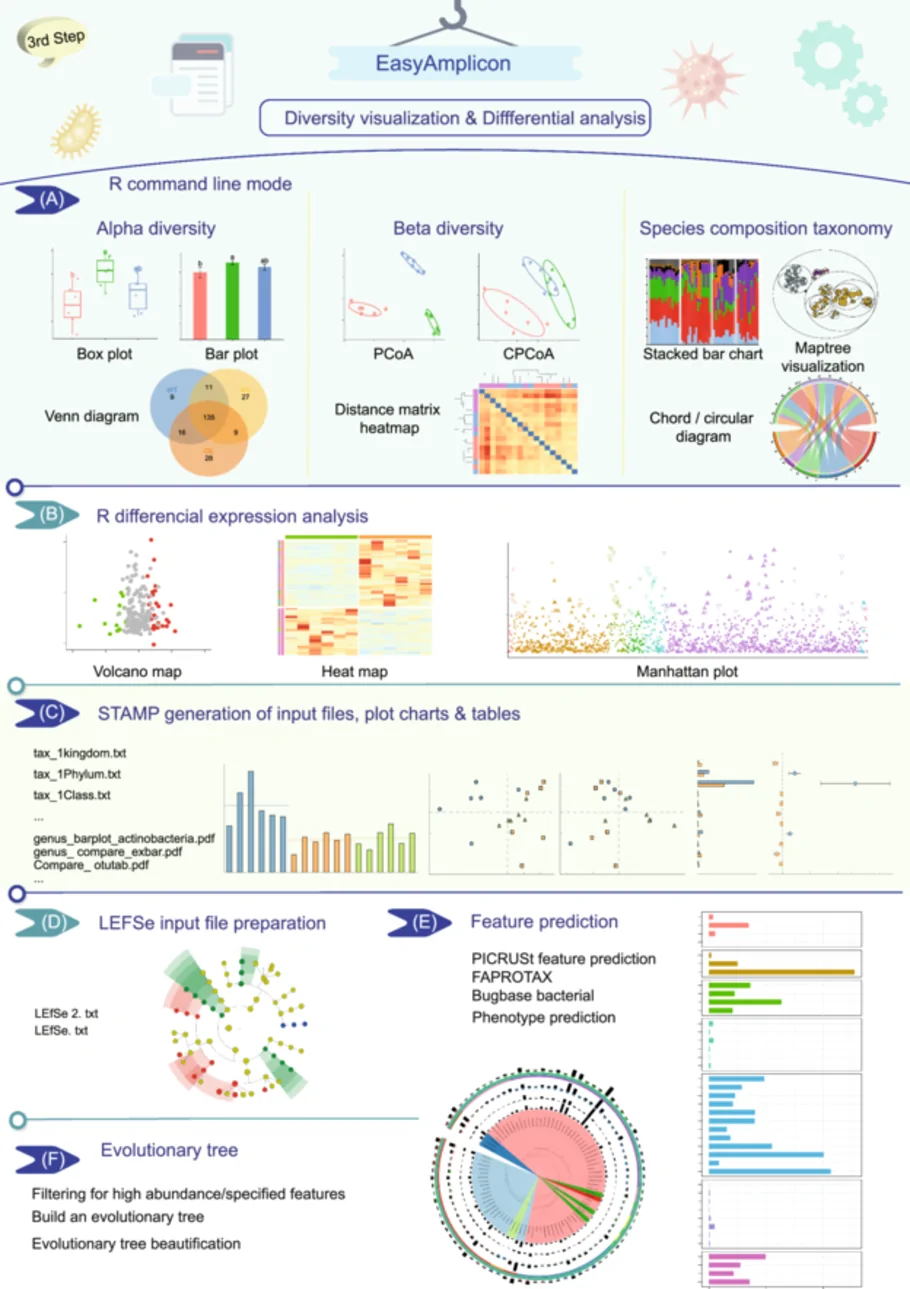
EasyAmplicon pipeline analyses offer diverse functions for analyzing amplicon sequencing data. (A) The pipeline delves into species diversity within a sample by generating visualizations such as boxplots, histograms, and Venn diagrams for α‐diversity. β‐diversity analysis explores the differences between microbial communities in various samples; this pipeline analyzes β‐diversity via distance matrix heat maps, principal coordinates analysis (PCoA), and canonical PCoA (CPCoA) plots. Moreover, the pipeline investigates the taxonomic makeup of a sample's microbial community through chord diagrams, stacked bar charts, and map tree visualizations for species composition and taxonomy. (B) The differential expression analysis visualizations include heat map, volcano plot, and Manhattan plot. (C, D) Beyond these core analyses, the pipeline integrates seamlessly with third‐party software by generating compatible input files for STAMP and LEfSe for in‐depth analysis. (E) It also offers comprehensive analysis of feature prediction with tools like PICRUSt, FAPROTAX, and BugBase to predict bacterial phenotypes, (F) and generates various plans to construct and beautify evolutionary trees using iTOL software.

EasyAmplicon pipeline offers functions to generate various alpha diversity plots, providing insights into the richness and evenness of microbial communities within each sample. These plots include alpha bar charts (typically displaying the number of observed OTUs), alpha boxplots (visualizing the distribution of OTU counts), and alpha rarefaction curves (estimating diversity at a common sequencing depth) [54]. Data files (e.g., alpha.txt, vegan.txt, alpha_rare.txt, otu_group_exist.txt) facilitate the generation of these plots using R packages like ggplot2 and vegan (Figure 4A).

##### 6.4.1.1 Boxplot

Run the following command to draw alpha boxplot:

Rscript ${db}/script/alpha_boxplot.R ‐h

Rscript ${db}/script/alpha_boxplot.R ‐‐alpha_index richness\

‐‐input result/alpha/vegan.txt ‐‐design result/metadata.txt\

‐‐group Group ‐‐output result/alpha/\

‐‐width 89 ‐‐height 59

To use loops to plot 6 commonly used indices, run this:

for i in 'head ‐n1 result/alpha/vegan.txt|cut ‐f 2‐';do

Rscript ${db}/script/alpha_boxplot.R ‐‐alpha_index ${i}\

‐‐input result/alpha/vegan.txt ‐‐design result/metadata.txt

‐‐group Group ‐‐output result/alpha/\

‐‐width 89 ‐‐height 59

done

mv alpha_boxplot_TukeyHSD.txt result/alpha/

Furthermore, to plot alpha diversity histogram and calculate standard deviations run the following commands:

Rscript ${db}/script/alpha_barplot.R ‐‐alpha_index richness\

‐‐input result/alpha/vegan.txt ‐‐design result/metadata.txt\

‐‐group Group ‐‐output result/alpha/

‐‐width 89 ‐‐height 59

##### 6.4.1.2 Dilution curve

The dilution curve illustrates the relationship between group sequencing depth mean and standard error with feature (OTU or ASV) counts. Using the input file alpha_rare.txt, specify the experimental design and grouping, set the output directory, and define the image dimensions (width and height). To draw a curve, run the following code:

Rscript ${db}/script/alpha_rare_curve.R\

‐‐input result/alpha/alpha_rare.txt ‐‐design result/metadata.txt

‐‐group Group ‐‐output result/alpha/\

‐‐width 120 ‐‐height 59

##### 6.4.1.3 Venn diagram

To generate a Venn diagram [55], select three groups for comparison. Use the input file “otu_group_exist.txt,” which contains two columns: ID and group. The command line options are as follows: “‐f” for the input file, “‐a/b/c/d/g” for group names, “‐w/u” for width and height in inches, and “‐p” for the output file name (default is the same as the input) (Figure 4A).

bash ${db}/script/sp_vennDiagram.sh

‐f result/alpha/otu_group_exist.txt\

‐a WT ‐b KO ‐c OE\

‐w 3 ‐u 3\

‐p WT_KO_OE

To compare four sets, run this code:

bash ${db}/script/sp_vennDiagram.sh

‐f result/alpha/otu_group_exist.txt\

‐a WT ‐b KO ‐c OE ‐d All\

‐w 3 ‐u 3\

‐p WT_KO_OE_All

**Method 2** (Drawing Alpha Boxplots using R Markdown (Rmd)): This section walks you through the process of analyzing alpha diversity using the provided R Markdown file (“Diversity.Rmd”) located in the “result” directory of EasyAmplicon master folder or the script directory of EasyMicrobiome. Open the Diversity.Rmd file in RStudio to run the code and generate visualizations. In the experimental design section of the Rmd file, carefully review the metadata related to your experiment. This involves verifying the grouping variable (group), image dimensions (width and height), and confirming the file and index type used for alpha diversity calculations, which defaults to “Richness.” There are two ways to execute the code: First, you can run the code line by line, allowing you to monitor the creation of each variable as the code progresses. Alternatively, you can execute all code blocks sequentially by using the right arrow button in RStudio or by “Knitting” the entire document, which will generate a comprehensive webpage report containing all results and calculations. The generated outputs can be found in the “alpha” directory. The boxplot for alpha diversity (richness) is located in the file “alpha/alpha_boxplot_Richness.pdf.” Users can also find the rarefaction curve () in the “alpha” directory, specifically in ‘alpha/alpha_rarefaction_curve.pdf’. Instructions within the # Rarefaction Curve section will provide details on input and output file locations for the specific visualization.

**Method 3:** Additionally, users can visit ImageGP (https://www.bic.ac.cn/BIC/#/), to draw box plot online (Figure S1A). First open data files (metadata.txt and vegan.txt) in excel and copy the “group” and “richness” columns into a new sheet. Paste this combined data into the website text box and select the grouping (group) and richness (*Y*‐axis). Click “PLOT” to generate the boxplot. Similarly, users can plot the Venn diagram and rarefaction curve using this link https://www.bic.ac.cn/BIC/#/ (Figure S1A).

#### 6.4.2 Beta diversity

##### 6.4.2.1 Distance matrix heatmap

Distance matrices are tables that represent the dissimilarity (difference) between samples based on their microbial composition. They play a crucial role in microbiome analysis for tasks like clustering similar samples. To draw a distance matrix heatmap using Bray–Curtis distance as an example, you can run the following code:

bash ${db}/script/sp_pheatmap.sh\

‐f result/beta/bray_curtis.txt\

‐H 'TRUE' ‐u 6 ‐v 5

To add grouping annotations (such as genotype and location) in columns 2 and 4, run this code:

cut ‐f 1‐2 result/metadata.txt > temp/group.txt

Followed by the following code to draw the heat map:

bash ${db}/script/sp_pheatmap.sh\

‐f result/beta/bray_curtis.txt\

‐H 'TRUE' ‐u 6.9 ‐v 5.6\

‐P temp/group.txt ‐Q temp/group.txt

Where “‐f” is the input file, “‐h” is clustering (TRUE/FALSE), “‐u/v” is the width and height (inches), “‐P” adds line annotations to the file, and “‐Q” adds column annotations (Figure 4A).

##### 6.4.2.2 Primary coordinate analysis PCoA

This section describes how to perform PCoA to visualize beta diversity patterns in data. There are two main methods:

**Method 1:** This method utilizes the provided R script “beta_pcoa.R,” run the script which used bray_curtis.txt and metadata.txt as an input file and generates output bray_curtis. cpcoa.pdf.

Rscript ${db}/script/beta_pcoa.R\

‐‐input result/beta/bray_curtis.txt ‐‐design result/metadata.txt

‐‐group Group ‐‐label FALSE ‐‐width 89 ‐‐height 59\

‐‐output result/beta/bray_curtis.pcoa.pdf

And to add sample labels with the parameter ‘‐‐label’ TRUE, run this script:

Rscript ${db}/script/beta_pcoa.R

‐‐input result/beta/bray_curtis.txt ‐‐design result/metadata.txt

‐‐group Group ‐‐label TRUE ‐‐width 89 ‐‐height 59\

‐‐output result/beta/bray_curtis.pcoa.label.pdf

mv beta_pcoa_stat.txt result/beta/

Run the following code to obtain the p‐values for pairwise comparisons between groups (note that the probability values may vary slightly with each execution).

cat result/beta/beta_pcoa_stat.txt

**Method 2:** Alternatively, users can perform PCoA analysis using the provided R Markdown file “Diversity.Rmd.” Open this file in RStudio and navigate to the section titled # β diversity —— ## Principal Axis Analysis. Knitting the document will execute the relevant code and generate the PCoA plot (beta/pcoa_bray_curtis.pdf) along with the beta_pcoa_stat.txt file (containing pairwise comparison *p* values) in the result/beta directory.

**Method 3**: For those who prefer a web‐based approach, can explore ImageGP (http://www.bic.ac.cn/ImageGP/) to generate PCoA plots and heat map (Figure S1A).

##### 6.4.2.3 Constrained principal coordinates analysis (CPCoA)

This section covers CPCoA, a technique that explores how environmental factors or other explanatory variables influence patterns of beta diversity. There are two main methods for performing CPCoA:

**Method 1:** This method utilizes the provided R script “beta_cpcoa.R.” The script arguments are similar to those used for the “beta_pcoa.R” script. For this, run the following code:

Rscript ${db}/script/beta_cpcoa.R\

‐‐input result/beta/bray_curtis.txt ‐‐design result/metadata.txt\

‐‐group Group ‐‐output result/beta/bray_curtis.cpcoa.pdf\

‐‐width 89 ‐‐height 59

Add sample labels with the parameter ‐‐label TRUE

Rscript ${db}/script/beta_cpcoa.R

‐‐input result/beta/bray_curtis.txt ‐‐design result/metadata.txt

‐‐group Group ‐‐label TRUE ‐‐width 89 ‐‐height 59\

‐‐output result/beta/bray_curtis.cpcoa.label.pdf

**Method 2:** Alternatively, user can perform CPCoA using the provided R Markdown file “Diversity.Rmd.” Open this file in RStudio and navigate to the section titled # βdiversity — ## supervised PCoA(CPCoA). The script within this section likely references example files paths. User can modify these paths to point to the location of actual beta diversity matrices (likely stored in the result/beta directory). Once the file paths are adjusted, knit the R Markdown document. This will generate a comprehensive webpage report containing CPCoA plots (including one for Bray‐Curtis distance) and other relevant results. An additional file named “beta/cpcoa_bray_curtis.pdf” will also be created, containing the CPCoA plot for Bray–Curtis distance specifically.

**Method 3:** While the provided script supports Bray–Curtis distance, if user wants to explore a wider range of distance metrics, consider the online tool http://www.bic.ac.cn/ImageGP/ (Figure S1A). This tool offers more than a dozen non‐evolutionary distance options, but cannot calculate Unifrac‐related distances. The best method choice (local script or online tool) depends on your specific needs. Consider the type of distance metric you want to visualize and your familiarity with R scripting.

#### 6.4.3 Species composition taxonomy

This pipeline empowers users to explore the taxonomic makeup of microbial communities across various levels (genus, family, phylum) through diverse visualization like stacked bar charts (stackplots), circular diagrams (chord diagrams), and tree maps (Figure 4A). There are two methods to draw each.

##### 6.4.3.1 Stackplot

**Method 1:** Taking the phylum (p) level as an example, the results will include two files: “output.sample.pdf” and “output.group.pdf.” The input file “sum_p. txt” generated earlier.

Rscript ${db}/script/tax_stackplot.R\

‐‐input result/tax/sum_p.txt ‐‐design result/metadata.txt

‐‐group Group ‐‐color ggplot ‐‐legend 7 ‐‐width 89 ‐‐height 59\

‐‐output result/tax/sum_p.stackplot

Colors can be modified using ggplot manual1 (22), Paired (12), or Set3 (12) color palettes.

Rscript ${db}/script/tax_stackplot.R\

‐‐input result/tax/sum_p.txt ‐‐design result/metadata.txt

‐‐group Group ‐‐color Paired ‐‐legend 12 ‐‐width 181 ‐‐height 119\

‐‐output result/tax/sum_p.stackplotPaired

To perform batch drawing, using input data that includes five taxonomic levels: phylum (p), class (c), order (o), family (f), and genus (g). Run this code:

for i in p c o f g; do

Rscript ${db}/script/tax_stackplot.R\

‐‐input result/tax/sum_${i}.txt ‐‐design result/metadata.txt\

‐‐group Group ‐‐output result/tax/sum_${i}.stackplot\

‐‐legend 8 ‐‐width 89 ‐‐height 59; done

**Method 2** (Stacked histogram drawing via command line/Rmd (optional)): Open Diversity.Rmd with Rstudio and locate the # Species Composition Taxonomy stackplot section, adjust taxonomy level, input and output file location and picture size, and just click the command to run.

##### 6.4.3.2 Chord/circular diagram using circlize

Taking class (c) as an example and to draw circular diagram with top 5 groups, run the following code:

i=c

Rscript ${db}/script/tax_circlize.R\

‐‐input result/tax/sum_${i}.txt ‐‐design result/metadata.txt\

‐‐group Group ‐‐legend 5

The results are located in the current directory: “circlize.pdf” (with random colors) and “circlize_legend.pdf” (with specified colors and legend). To ensure consistency with the classification level, move and rename the files by executing the following code:

mv circlize.pdf result/tax/sum_${i}.circlize.pdf

mv circlize_legend.pdf result/tax/sum_${i}.circlize_legend.pdf

**Method 2** (Chord/circular diagram via Rmd): Alternatively, open “Diversity.Rmd” with Rstudio and navigate to the ## chord diagram circlize plot section. Adjust the classification level, the number of legends, and the grouping column names as needed. Right‐click to run the paragraph, and view the resulting circlize. pdf and circlize_legend.pdf in the directory.

##### 6.4.3.3 Treemap visualization

To create a hierarchical tree map visualization depicting species relationships, run the following code. The code uses the input feature table and species annotations to generate the treemap. Be sure to specify the number of features and the image dimensions.

Rscript ${db}/script/tax_maptree.R\

‐‐input result/otutab.txt ‐‐taxonomy result/taxonomy.txt

‐‐output result/tax/tax_maptree.pdf\

‐‐topN 100 ‐‐width 183 ‐‐height 118

#### 6.4.4 R differential analysis

Video tutorial link: https://youtu.be/FsdYNQ5DtHg

##### 6.4.4.1 Comparison analysis

This step focuses on identifying features that differ significantly between groups. To analyze these differences, the user must specify input data, define the comparison group, run the statistical test, set parameters, and generate output as a volcano plot, heat map, Manhattan plot, individual feature drawing, and ternary diagram (Figure 4B). First, create a directory named “compare” in the result folder by running the following code “mkdir ‐p result/compare/.” Use the input feature table (otutab.txt) and metadata.txt to specify the grouping column name, comparison groups, and abundance. Select the edgeR method with a *p*‐value threshold of 0.05 and FDR threshold of 0.2 for the output directories. Run the following code:

compare = “KO‐WT"

Rscript ${db}/script/compare.R\

‐‐input result/otutab.txt ‐‐design result/metadata.txt\

‐‐group Group ‐‐compare ${compare} ‐‐threshold 0.1\

‐‐method edgeR ‐‐pvalue 0.05 ‐‐fdr 0.2\

‐‐output result/compare/

▲**Troubleshooting:** If you encounter the error “Error in file (file, ifelse(append, ‘a,’ ‘w’)): Unable to open link Calls: write.table ‐> file.” It may indicate that the output directory does not exist. Ensure you create the directory as mentioned above.

##### 6.4.4.2 Visualization and analysis of differential abundance results

The EasyAmplicon pipeline offers various functionalities, especially this section involves generating visualizations to explore the differential abundance results.

**Volcano map**

To plot the volcano map (Figure 4B) and visualize the relationship between fold change and statistical significance for differentially abundant features, run the following code:

Rscript ${db}/script/compare_volcano.R\

‐‐input result/compare/${compare}.txt\

‐‐output result/compare/${compare}.volcano.pdf

‐‐width 89 ‐‐height 59

**Heat map**

To draw a heatmap displaying the abundance patterns of features across samples and groups, run the code below, which filters the number of columns, specifies metadata and grouping, and set species annotations, figure size, and font size (Figure 4B).

bash ${db}/script/compare_heatmap.sh ‐i

result/compare/${compare}.txt ‐l 7\

‐d result/metadata.txt ‐A Group

‐t result/taxonomy.txt\

‐w 8 ‐h 5 ‐s 7\

‐o result/compare/${compare}

**Map of Manhattan**

This section explains how to generate Manhattan plot to visualize statistically significant differences in species abundance between groups (Figure 4B). These plots are helpful for identifying key features that distinguish samples. To draw differential composition, run the following code:

bash ${db}/script/compare_manhattan.sh ‐i

result/compare/${compare}.txt

‐t result/taxonomy.txt\

‐p result/tax/sum_p.txt\

‐w 183 ‐v 59 ‐s 7 ‐l 10\

‐o result/compare/${compare}.manhattan.p.pdf

Here, “i” represents the difference comparison results, “t” represents species annotation, “p” represents legend, “w” represents width, “v” represents height, “s” represents size, and “l” represents legend maximum. To show the details, switch to class c and ‐L class by using this code:

bash ${db}/script/compare_manhattan.sh ‐i result/compare/${compare}.txt

‐t result/taxonomy.txt\

‐p result/tax/sum_c.txt\

‐w 183 ‐v 59 ‐s 7 ‐l 10 ‐L Class

‐o result/compare/${compare}.manhattan.c.pdf

To show the full legend, run the following code:

bash ${db}/script/compare_manhattan.sh ‐i result/compare/${compare}.txt\

‐t result/taxonomy.txt\

‐p result/tax/sum_c.txt\

‐w 183 ‐v 149 ‐s 7 ‐l 10 ‐L Class

‐o result/compare/${compare}.manhattan.c.legend.pdf

Note: Alternatively, users can use the ImageGP (http://www.bic.ac.cn/ImageGP/) to plot volcano map, heat map, and manhattan plot (Figure S1A).

**Drawing of individual features**

This step focuses on visualizing and analyzing the differential ASVs and OTUs identified in the previous steps. To screen and display the differentially abundant ASVs in the KO group, run the following code:

awk '$4 < 0.05' result/compare/KO‐WT.txt | sort ‐k7,7nr | cut ‐f1 | head

▲**Troubleshooting:** If the “awk” command returns no results (empty output), it might indicate a lack of differentially abundant ASVs (*p*‐value > 0.05) in the data. You can adjust the significance threshold (0.05) or investigate potential issues with the data or comparison groups.

**Differential OTU Analysis**: To display differential OTU detail, run the following command:

Rscript ${db}/script/alpha_boxplot.R ‐‐alpha_index ASV_2 ‐‐input result/otutab.txt ‐‐design result/metadata.txt ‐‐transpose TRUE ‐‐scale TRUE ‐‐width 89 ‐‐height 59 ‐‐group Group ‐‐output result/compare/feature_

The script “Rscript ${db}/script/alpha_boxplot.R” launches an R script named alpha_boxplot.R located in the “${db}/script” directory. This script generates a boxplot visualization comparing the abundance of a specific alpha diversity index (ASV_2) across different groups defined in the “metadata.txt” file. Additional arguments within the script control output formatting and dimensions.

▲**Troubleshooting:** This error “Error: data. frame(…).” indicates an issue with the number of rows in the data being combined for the boxplot. This could happen if the “otutab.txt” or “metadata.txt” files have different numbers of rows for each sample. Check for inconsistencies in the data processing or filtering steps and ensure that both files have matching sample information.

**Genus‐Level Analysis:** To sort columns in descending order by mean genus abundance, run the following command:

csvtk ‐t sort ‐k All:nr result/tax/sum_g.txt | head

This code utilizes the “csvtk” tool to sort the “sum_g.txt” file located in the “result/tax” directory. The “‐t” flag specifies a tab‐delimited file, “‐k All:nr” sorts all columns numerically in descending order, and “head” displays the top lines (showing the most abundant genera). To further explore the details of attribute‐level differences, run the following code:

Rscript ${db}/script/alpha_boxplot.R ‐‐alpha_index Lysobacter\

‐‐input result/tax/sum_g.txt ‐‐design result/metadata.txt\

‐‐transpose TRUE\

‐‐width 89 ‐‐height 59\

‐‐group Group ‐‐output result/compare/feature_

▲**Troubleshooting:** If the boxplot generated from the genus abundance data appears uninformative (poor details), it might be due to a high number of genera. Consider filtering the data to focus on the top abundant genera or using alternative visualization methods like bar charts.

#### 6.4.5 Differential abundance analysis using STAMP

Video tutorial link: https://youtu.be/PptGLAI93eE

STAMP is a separate software package for analyzing microbiome data. This section provides an overview of using STAMP for differential abundance analysis (Figure 4C).

##### 6.4.5.1 Generate an input file

These steps involve formatting data for use in STAMP and generating visualizations of the differential abundance results.

Rscript ${db}/script/format2stamp.R ‐h

mkdir ‐p result/stamp

Rscript ${db}/script/format2stamp.R ‐‐input result/otutab.txt ‐‐taxonomy result/taxonomy.txt ‐‐threshold 0.01 ‐‐output result/stamp/tax

The output results are located in the results/stamp directory. Users can open these files in the STAMP software. The results include files named tax_1 to tax_8. tax_1 to tax_7 is the classification summary of kingdom, phylum, class, order, family, genus and species and tax_8 is the filtered OTU table, where, for example, 0.01 represents the average filtered abundance. This pipeline also offers an alternative method for generating the input files through format2stamp. Rmd, located in the result directory of the EasyAmplicon master folder or within the script directory of EasyMicrobiome database folder. To use format2stamp.Rmd, users must have “otutab.txt” and “taxonomy.txt” files in the result directory. Next, open “format2stamp.Rmd” with Rstudio. Before running the code, review and adjust any settings within the code, such as input and output file names, OTU/ASV relative abundance filtering thresholds, as needed. Click the “Knit” button in RStudio to execute the code. By default, the result will be saved in the result/stamp directory.

##### 6.4.5.2 Plot extended column charts and tables

To compare the two groups (e.g., KO‐WT), it is recommended to use Welch's *t*‐test along with an extended error bar or heatmap. Replace the ASV data in result/otutab.txt with genus data from result/tax/sum_g.txt, and run the following command:

compare=“KO‐WT"

Rscript ${db}/script/compare_stamp.R\

‐‐input result/stamp/tax_5Family.txt ‐‐metadata result/metadata.txt\

‐‐group Group ‐‐compare ${compare} ‐‐threshold 0.1\

‐‐method "t.test" ‐‐pvalue 0.05 ‐‐fdr "none"\

‐‐width 189 ‐‐height 159\

‐‐output result/stamp/${compare}

An optional method is also available through “compareStamp. Rmd,” which can be found in the result directory of EasyAmplicon master folder or in the script directory of EasyMicrobiome folder. This file contains code that users can review and adjust according to their needs, including the names and locations of input and output files or specific thresholds. Once you are satisfied with the settings, click the “Knit” button in RStudio to execute the code, generating the required input files. By default, the results will be placed in the “result/compare” directory.

Note: It is recommended to use PCA, ANOVA + FDR + single feature boxplot for multi‐group comparison.

#### 6.4.6 Generation of input files for LEfSe

Video tutorial link: https://youtu.be/VXJvdidyFlU

LEfSe (Linear discriminant analysis Effect Size) analysis is used to identifying microbial features that differ significantly between groups. This section provides an overview of two methods used to generate the input files for LEfSe analysis (Figure 4D).

**Method 1:** In this method, run the following command to generate input files. The threshold parameter regulates abundance filtering, allowing you to control the number of branches in the map.

mkdir ‐p result/lefse

Rscript ${db}/script/format2lefse.R ‐‐input result/otutab.txt ‐‐taxonomy result/taxonomy.txt ‐‐design result/metadata.txt ‐‐group Group ‐‐threshold 0.4 ‐‐output result/lefse/LEfSe

**Method 2** (optional): Alternatively, user can generate input file via using format2lefse.Rmd file. The result directory contains three files: otutab.txt, metadata.txt, taxonomy.txt. To proceed, navigate to the db/script directory, locate the format2lefse. Rmd file, and open it with RStudio. Running the code will generate two input files: LEfSe.txt and LEfSe2.txt file. For a comprehensive LEfSe analysis and visualization, we recommend two online tools. First, use the ImageGP online server (https://www.bic.ac.cn/BIC/#/analysis?page=b%27MzY%3D%27). Simply upload your LEfSe input file (LEfSe.txt), submit it, and within approximately 3 min, you'll receive high‐quality visualizations of your LEfSe results (Figure S1B). Alternatively, the LEfSe website (https://huttenhower.sph.harvard.edu/lefse/) offers a web‐based platform for LEfSe analysis.

**Method 3:** LEfSe local analysis under Linux is optional and explains in separate section titled “additional analyses under Linux.”

#### 6.4.7 Feature prediction analysis

This section describes how to predict functional and morphological features of microbial communities using PICRUSt and Bugbase, respectively. Feature prediction, in this context, refers to the process of inferring functional or morphological characteristics of microbes based on their marker gene sequences obtained from amplicon sequencing.

##### 6.4.7.1 PICRUSt feature prediction

Video tutorial link: https://youtu.be/bq9IkEJVAoQ

PICRUSt (Phylogenetic Investigation of Communities by Reconstruction of Unobserved States) is an analysis tool specifically designed to predict the functional capabilities (e.g., metabolic pathways) of a microbial community based on marker gene data, typically 16S rDNA gene sequences [29, 56]. It leverages pre‐existing knowledge about the relationship between marker gene sequences and functional genes found in sequenced genomes (Figure 4E).

###### 6.4.7.1.1 PICRUSt 1.0

**Method 1** (Command‐line analysis runs locally on Windows): To predict features, first step is the file preparation, which involves generating an OTU table by comparing the Greengenes database [40]. This has already been done above (see pipeline. sh/pipeline_eng.sh, step 9 for reference), resulting in the “otutab.txt” file. Second step is to use online webserver ImageGP (https://www.bic.ac.cn/BIC/#/) to generate key files by uploading the gg/otutab.txt file. Ensure to modify the OUT ID to “OTUID” before uploading. The results generated have four levels (Figure S1C), download all the output files and save them in the working directory path EasyAmplicon/result/picrust (generate picrust folder in result directory). The obtained files can be directly transferred to STAMP for visualization, as STAMP is a powerful tool for integrating and displaying detailed analysis results at all levels [57].

Now, run the following code to remove the extra detail from the files downloaded from ImageGP in order to align file format with the subsequent analysis.

cd ${wd}/result/picrust/

find. ‐type f ‐name "*all_level*" ‐exec bash ‐c 'mv "$0" "${0/*all_level/all_level}"' {}\;

cd ${wd}

Next, run the code given below:

l = L2

sed '/# Const/d;s/OTU//' result/picrust/all_level.ko.${l}.txt > result/picrust/${l}.txt

num = 'head ‐n1 result/picrust/${l}.txt|wc ‐w'

paste <(cut ‐f $num result/picrust/${l}.txt) <(cut ‐f 1‐$[num‐1] result/picrust/${l}.txt)

> result/picrust/${l}.spf

cut ‐f 2‐ result/picrust/${l}.spf > result/picrust/${l}.mat.txt

awk 'BEGIN{FS=OFS=“\t"} {print $2,$1}' result/picrust/${l}.spf | sed 's/;/\t/' | sed '1s/ID/Pathway\tCategory/'

> result/picrust/${l}.anno.txt

To visualize the KEGG hierarchical function histogram, execute the following code. The generated result file will serve as the input for visualizing the abundance distribution map.

compare=“KO‐WT"

Rscript ${db}/script/compare.R ‐‐input result/picrust/${l}.mat.txt ‐‐design result/metadata.txt ‐‐group Group ‐‐compare ${compare} ‐‐threshold 0 ‐‐method wilcox ‐‐pvalue 0.05 ‐‐fdr 0.2 ‐‐output result/picrust/

Then, to draw an abundance distribution map of microbial communities based on KEGG prediction functions, the result ${compare} can be filtered into a ‘. txt file’. Execute the following code to generate a bar plot (histogram):

Rscript ${db}/script/compare_hierarchy_facet.R ‐‐input result/picrust/${compare}.txt ‐‐data MeanA ‐‐annotation result/picrust/${l}.anno.txt ‐‐output result/picrust/${compare}.MeanA.bar.pdf

Following to generate bar plots depicting significant differences between two groups, with faceting based on higher taxonomic levels, run this code:

Rscript ${db}/script/compare_hierarchy_facet2.R\

‐‐input result/picrust/${compare}.txt\

‐‐pvalue 0.05 ‐‐fdr 0.1\

‐‐annotation result/picrust/${l}.anno.txt\

‐‐output result/picrust/${compare}.bar.pdf

**Method 2** (Local operation‐limited to Linux system, install picrust environment): First, launch the Linux subsystem and install Conda. Next, use “conda install picrust” to set up the software environment for feature prediction. To ensure compatibility with downstream analyses, convert OTU table to a common format. This method explain in detail under the section “Analysis under Linux System.”

##### 6.4.7.2 FAPROTAX

Video tutorial links: https://youtu.be/kAiMq08mLNs

The Functional Annotation of prokaryotic taxa (FAPROTAX) database (http://www.loucalab.com/archive/FAPROTAX) offers a user‐friendly approach to predicting potential functions based on the 16S rRNA gene amplicon data [32]. It provides a freely available python script (collapse_table.py) that translates taxonomic profiles, like OTU tables, into potential functional profiles. FAPROTAX leverages a comprehensive database containing over 7600 functional annotations for more than 4600 taxa across 80+ functions, encompassing metabolic pathways and ecologically relevant processes like nitrification, de‐nitrification, and fermentation. This resource maps specific prokaryotic taxa (e.g., genera or species) to functional roles, aiding researchers to uncover the metabolic potential of microbial communities [58, 59].

**Method 1:** ImageGP offers a one‐click, web‐based platform for functional annotation of microbial communities using FAPROTAX. This simplifies the process by eliminating complex command‐line work or software installations. Upload the OTU table to the ImageGP online webserver at (https://www.bic.ac.cn/BIC/#/), which will handle the FAPROTAX analysis (Figure S1D), offering comprehensive results and visualizations.

**Method 2** (Under Linux system‐optional): For users who prefer or require a local analysis, the FAPROTAX pipeline can be executed on a Linux system. This method utilizes command‐line tools and locally installed databases. Details can be found in the “Analysis under Linux System” section. This approach provides flexibility for experienced Linux users, allowing them to customize their analysis pipeline as needed.

##### 6.4.7.3 Bugbase bacterial phenotype prediction

Video tutorial link: https://youtu.be/OvkZxjOjwmE

Bugbase is a tool for predicting bacterial phenotypes from microbiome samples [31]. It allows for the prediction and comparison of various bacterial characteristics based on OTU tables and mapping files, including the following seven aspects: Gram‐positive, Gram‐negative, biofilm forming, pathogenic potential, mobile element containing, oxygen utilizing, and oxidative stress tolerant. Typically, Bugbase is used to predict the relative abundance of Gram‐positive/negative bacteria, as well as stress‐tolerant and pathogenic bacteria in microbiome samples based on 16S rRNA data.

**Method 1** (Run locally on Windows): Code and packages were updated based on R 4.x. Specify the software directory as the bugbase variable in pipeline, enter the gg OTU table, metadata, and specify the group column name and output directory.

cd ${wd}/result

bugbase=${db}/script/BugBase

rm ‐rf bugbase/

Rscript ${bugbase}/bin/run.bugbase.r ‐L ${bugbase} ‐i gg/otutab.txt ‐m metadata.txt ‐c Group ‐o bugbase/

**Method 2:** Alternatively, users can use online web server to perform Bugbase analysis using ImageGP (http://www.bic.ac.cn/ImageGP/index.php/Home/Index/BugBase.html) (Figure S1E). Additionally, users can access the Bugbase website at https://bugbase.cs.umn.edu/. However, it is not recommended due to its susceptibility to errors.

**Method 3** (Installation and use in Linux system‐optional): Detailed instructions for Bugbase installation and use under a Linux system are provided in the section titled “Analysis under Linux System.”

#### 6.4.8 Construction of advance phylogenetic tree

Video tutorial link: https://youtu.be/c3cM-zwbChs

This section explains how to construct and visualize phylogenetic tree [60] with various schemes. A new folder named “tree” is created within the “result” folder to store generated files (Figure 4F) by executing the following code:

cd ${wd}

mkdir ‐p result/tree

cd ${wd}/result/tree

##### 6.4.8.1 Filtering high abundance/specified features

**Method 1:** Filter features based on abundance, typically selecting 0.001 or 0.005, with the OTU count falling within the range of 30 to 150. Count the number of ASVs in the feature table using the code “tail ‐n+2../otutab_rare.txt | wc ‐l.” Then, filter high‐abundance OTUs based on a relative abundance threshold of 0.2% using this code:

usearch ‐otutab_trim../otutab_rare. txt ‐min_otu_freq. 0.002 ‐output otutab. txt

Next, count the number of features in the filtered OTU table using this command:

tail −n+2 otutab. txt | wc −l

**Method 2:** Filter based on quantity/count: Sort by abundance, defaulting to descending order (from largest to smallest).

usearch ‐otutab_sortotus../otutab_rare.txt

‐output otutab_sort.txt

Extract the OTU IDs from the top specified high‐abundance OTUs, such as the top 100, by running this code:

sed '1s/#OTU ID/OTUID/' otutab_sort.txt

| head ‐n101 > otutab.txt

Run the code “sed ‐i '1s/#OTU ID/OTUID/' otutab.txt” to modify the column name of the feature ID. To extract IDs for sequence retrieval, run this code:

cut ‐f 1 otutab.txt > otutab_high.id

Then, filter high‐abundance bacteria/specify differential bacteria‐associated OTU sequences by running this code:

usearch ‐fastx_getseqs../otus.fa ‐labels otutab_high.id ‐fastaout otus.fa

head ‐n 2 otus.fa

Filter OTUs based on species annotation by using the following command:

awk 'NR==FNR{a[$1]=$0} NR>FNR{print a[$1]}'../taxonomy. txt

otutab_high.id > otutab_high.tax

Obtain group mean values corresponding to OTUs for use in generating a sample heatmap. Dependent on the previously calculated group mean values using the “otu_mean.R” script.

awk 'NR==FNR{a[$1]=$0} NR>FNR{print a[$1]}'../otutab_mean. txt otutab_high.id

| sed 's/#OTU ID/OTUID/' > otutab_high.mean

Merge species annotation and abundance into an annotation file.

cut ‐f 2‐ otutab_high.mean > temp

paste otutab_high. tax temp > annotation. txt

head ‐n 3 annotation.txt

##### 6.4.8.2 Build an evolutionary tree

The starting files are the otus.fa (sequence), annotation.txt (species and relative abundance) in the result/tree directory. Use this command “muscle ‐in otus.fa ‐out otus_aligned.fas,” for sequence alignment.

**Method 1:** Use IQ‐TREE [61] database to quickly build ML evolutionary tree.

rm ‐rf iqtree

mkdir ‐p iqtree

iqtree ‐s otus_aligned. fas ‐bb 1000 ‐redo ‐alrt 1000 ‐nt AUTO ‐pre iqtree/otus

**Method 2:** Use “apt install fasttree” on Ubuntu to install FastTree, ideal for large phylogenetic trees (hundreds of OTUs). Build the tree by executing the code “fasttree ‐gtr ‐nt otus_aligned.fas > otus.nwk.”

##### 6.4.8.3 Evolutionary tree beautification

This section outlines the initial process for customizing the appearance of the phylogenetic tree. This pipeline focuses on coloring and shaping the outer circle based on species annotation and abundance information. Previously, we generated annotation.txt file, which contains a mapping between OTU identifiers and their corresponding species annotations and abundance values. Several options can be used such as:

**Scheme** **1.** For color and shape classification, as well as abundance scheme annotation, execute the following command:

cd ${wd}/result/tree

Rscript ${db}/script/table2itol.R ‐a ‐c double ‐D plan1 ‐i OTUID ‐l Genus ‐t %s ‐w 0.5 annotation.txt

**Scheme** **2.** To generate abundance histogram annotation files, run the following command:

Rscript ${db}/script/table2itol.R ‐a ‐d ‐c none ‐D plan2 ‐b Phylum ‐i OTUID ‐l Genus ‐t %s ‐w 0.5 annotation.txt

**Scheme** **3.** To generate heatmap annotation files, use the following command:

Rscript ${db}/script/table 2itol.R ‐c keep ‐D plan3 ‐i OTUID ‐t %s otutab.txt

**Scheme** **4.** To convert integers into factors and generate annotation files, run this code:

Rscript ${db}/script/table2itol.R ‐a ‐c factor ‐D plan4 ‐i OTUID ‐l Genus ‐t %s ‐w 0 annotation.txt

After generating the tree files, access iTOL website [34] at http://itol.embl.de/ and create an account. Navigate to the “otus.contree” file in the working directory EasyAmplicon/result/tree/iqtree folder, drag and drop it onto the iTOL uploads area. Next, upload the plan files individually for further customization, such as highlighting specific branches. iTOL offers various advance options to adjust the tree appearance—explore these features to beautify the tree according to the preferences. Once satisfied, save your customized tree within iTOL (Figure 4F). Alternatively, online visualization platform (https://www.bic.ac.cn/BIC/#/) is available, which offers a user‐friendly interface for visualizing evolutionary trees.

### 6.5 Additional analyses under Linux (Optional)

This section of the pipeline provides instructions for running analyses such as LEfSe, FAPROTAX, and PICRUSt functions locally on Linux servers. Users familiar with command‐line operations will find these steps straightforward. However, specific software installations are required, including a Linux system, the conda package, and QIIME2. While PICRUSt2 is an optional tool for predicting metagenome functional content, it is important to note that it is resource‐intensive. Before beginning, ensure that your terminal is switched from “Git Bash” to “Windows Subsystem for Linux.” Keep in mind that running analyses on Linux is resource‐intensive and may require a server environment with sufficient memory (>16GB). For visualizing LEfSe analysis, the use of the online web server is recommended as previously mentioned.

#### 6.5.1 LEfSe analysis

Linear Discriminant Analysis Effect Size (LEfSe, LDA Effect Size) is a tool for high‐dimensional biomarker mining to identify genomic features (such as genes, pathways, and taxonomies) that significantly characterize two or more groups in microbiome data [62, 63]. To begin the analysis, first create a directory for storing LEfSe outputs by running the following command:

mkdir ‐p ~/EasyAmplicon/lefse

cd ~/EAsyAmplicon/lefse

Next, install LEfSe (if not installed before) under window Linux subsystem by running this code conda install lefse. Subsequently, run this code lefse_format_input.py LEfSe.txt sample.in ‐u 1 ‐c 1 ‐o 1000000, which converts data into the LEfSe internal format. Adjust 1000000 to specify the maximum number of features to analyze. Run lefse by running the code run_lefse.py input.in input.res. Next, draw a species tree to annotate the differences by executing this code:

lefse_plot_cladogram.py input.res cladogram.pdf ‐‐format pdf

Similarly, to draw a histogram of all differential features, use the following command:

lefse_plot_res.py input.res res.pdf ‐‐format pdf

As well as, to draw a histogram of a single feature, similar to a barplot in STAMP, use the following command:

head input.res

lefse_plot_features.py ‐f one ‐‐feature_name "Bacteria.Firmicutes.Bacilli.Bacillales.Planococcaceae.Paenisporosarcina"

‐‐format pdf sample.in input.res Bacilli.pdf

Additionally, to draw all difference feature histograms in batches, users can run this code:

mkdir ‐p features

lefse_plot_features.py ‐f diff ‐‐archive none ‐‐format pdf\

sample.in input.res features/

#### 6.5.2 PICRUSt function prediction

PICRUSt predicts the functional capabilities of microbial communities based on marker gene sequences. For users with access to Linux servers, the following steps outline the local execution of PICRUSt functions. Initially, set the database directory to download the pre‐calculated table from the specified URL.

db=/mnt/c/EasyMicrobiome

cd $db/gg

wget ‐c http://www.imeta.science/db/amplicon/GreenGenes/ko_13_5_precalculated.tab.gz

The next step involves setting up the Conda environment. To create and activate the Conda environment, execute the following code:

n=picrust

conda create ‐n ${n} ${n} ‐c bioconda ‐y

wd= /mnt/c/EasyAmplicon

cd $wd/result/gg

conda activate picrust

Next step is data preparation. Navigate to the directory containing the processed OTU table (otutab.txt) in Greengenes format. Convert the OTU table to BIOM format using the biom convert command by running the following code:

biom convert ‐i otutab.txt\

‐o otutab.biom\

‐‐table‐type=“OTU table" ‐‐to‐json

To perform function prediction, define the database directory (e.g.,/mnt/c/db) containing PICRUSt reference files. To normalize the OTU table by copy number, run the following code:

db= /mnt/c/EasyMicrobiome

normalize_by_copy_number.py ‐i otutab.biom\

‐o otutab_norm.biom\

‐c ${db}/gg/16S_13_5_precalculated.tab.gz

To predict metagenomic KEGG Orthology (KO) table, run the following code which generates a BIOM format file (ko.biom) (suitable for downstream analysis) and tab‐delimited text file (ko.txt) (for viewing and simpler analysis). Here ko_13_5_precalculated.tab.gz database is used to predict metagenomic table.

predict_metagenomes.py ‐i otutab_norm.biom

‐o ko.biom\

‐c ${db}/gg/ko_13_5_precalculated.tab.gz

predict_metagenomes.py ‐f ‐i otutab_norm.biom

‐o ko.txt\

‐c ${db}/gg/ko_13_5_precalculated.tab.gz

To classify and summarize function by level, process ko.txt for downstream analysis and run the following code:

sed ‐i '/# Constru/d;s/#OTU//' ko.txt

num= 'head ‐n1 ko.txt|wc ‐w'

paste <(cut ‐f $num ko.txt) <(cut ‐f 1‐$[num‐1] ko.txt) > ko.spf

for i in 1 2 3; do

categorize_by_function.py ‐f ‐i ko.biom ‐c KEGG_Pathways ‐l ${i} ‐o pathway${i}.txt

sed ‐i '/# Const/d;s/#OTU//' pathway${i}.txt

paste <(cut ‐f $num pathway${i}.txt) <(cut ‐f 1‐$[num‐1] pathway${i}.txt) > pathway${i}.spf

done

wc ‐l *.spf

#### 6.5.3 FAPROTAX

This section outlines the steps involved in performing FAPROTAX analysis using the EasyAmplicon pipeline. The required input files include an OTU table in txt format (optional, biom format preferred) and taxonomy file associated with the OTU table. The output will be; faprotax.txt (functional prediction results for each OUT), faprotax_report.txt (detailed report on OTU assignments to functional categories), faprotax_report.mat (matrix summarizing functional annotations). The first step is to create a working directory. The script directory “sd” should point to the location where the FAPROTAX scripts are stored within the database directory. Run this code:

wd=/mnt/c/EasyAmplicon/result/faprotax/

mkdir ‐p ${wd} && cd ${wd}

sd=/mnt/c/EasyMicrobiome/script/FAPROTAX_1.2.10

Second, is software installation, FAPROTAX dependencies might be satisfied within a QIIME 2 environment. Activate the appropriate QIIME 2 environment (refer to QIIME 2 part for activation instructions). To determine the FAPRATOX functionality and to check if it is installed correctly, run this code:

Python $sd/collapse_table.py

Next, prepare the input OTU table and convert it to the biom format by running the following command:

biom convert ‐i../otutab_rare.txt ‐o otutab_rare.biom ‐‐table‐type = “OTU table" ‐‐to‐json

To add species annotations to the biom table, run the following code:

biom add‐metadata ‐i otutab_rare.biom ‐‐observation‐metadata‐fp../taxonomy2.txt\

‐o otutab_rare_tax.biom ‐‐sc‐separated taxonomy ‐‐observation‐header OTUID,taxonomy

To find FAPROTAX functional prediction, run the code given below:

python ${sd}/collapse_table.py ‐i otutab_rare_tax.biom

‐g ${sd}/FAPROTAX.txt\

‐‐collapse_by_metadata 'taxonomy' ‐v ‐‐force\

‐o faprotax.txt ‐r faprotax_report.txt

Finally, to generate functional annotation matrix, run this code:

grep 'ASV_' ‐B 1 faprotax_report.txt | grep ‐v ‐P '^‐‐$' > faprotax_report.clean

**Figure 5:**
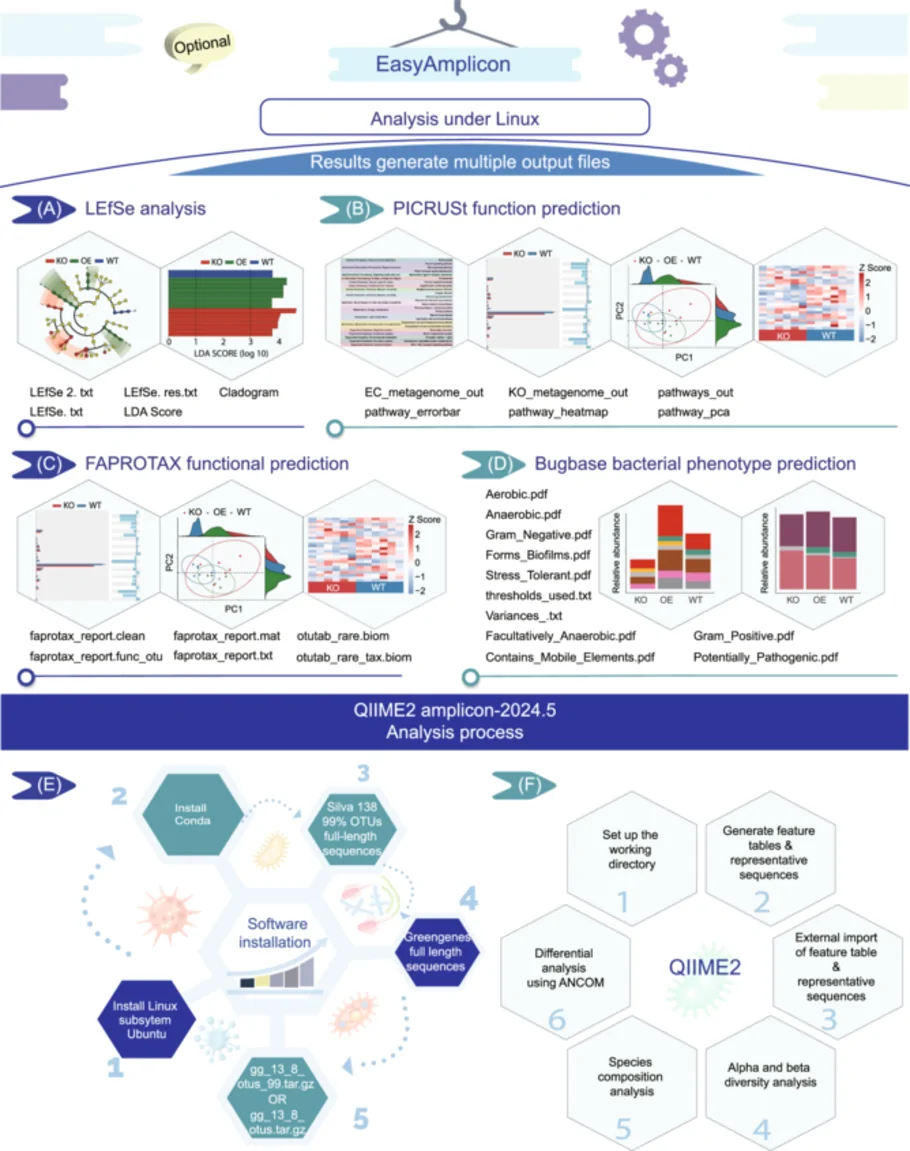
EasyAmplicon analyses under the Linux system and analyses integrated with QIIME 2 pipeline. (A) LEfSe (linear discriminant analysis effect size), a microbiome analysis method for identifying biomarkers that are differentially abundant between groups. LEfSe analysis utilizes input files such as LEfSe.txt, LEfSe.res.txt, and otutab_rare.biom. (B) PICRUSt functional prediction predicts the functional composition of a microbial community from marker gene sequencing data, outputting files like EC_metagenome_out, KO_metagenome_out, and pathways_out. (C) FAPROTAX functional Prediction infers functional profiles of microbial communities from marker gene data, generating outputs including faptotax_report.txt, faptotax_report.func_otu, and faptotax_report.mat. (D) BugBase, a bacterial phenotype prediction tool, analyzes microbiome data to predict bacterial phenotypes, producing various PDFs and text files such as aerobic.pdf, anaerobic.pdf, forms_biofilms.pdf, gram_negative.pdf, gram_positive.pdf, facultatively_anaerobic. pdf, contains_mobile_elements.pdf, potentially_pathogenic.pdf, stress_tolerant.pdf, thresholds_used.txt, and variances. txt. (E) The QIIME 2 analysis setup begins with the installation and activation of Conda and various databases, including Silva 138 99% and Greengenes. (F) The QIIME 2 analysis workflow starts by setting up the working directory. Next, it generates feature tables that summarize the abundance of biological features in samples, with representative sequences chosen to represent each operational taxonomic unit (OTU). Optional external import allows users to import data generated from other software into QIIME 2 for further analysis. Then, alpha and beta diversity analyses are utilized to assess the diversity within and between microbial communities. Following this, species annotation uses a reference database like Greengenes to assign taxonomic information to OTUs or ASVs in the feature table. Finally, differential abundance analysis uses ANCOM to identify features that are differentially abundant between groups of samples. ASVs, amplicon sequence variants.

Note: FAPROTAX is typically pre‐downloaded within the EasyAmplicon script directory. If a newer version is desired (e.g., version 1.2.10 used here), download it and place it in the script directory. Make sure to replace path to EasyMicrobiome/script/FAPROTAX_1.2. XX with the actual path on your system.

The output results generated against this analysis are listed in Figure 5A.

#### 6.5.4 Bugbase bacterial phenotype prediction

This section details the steps involved in performing Bugbase bacterial phenotype prediction using the EasyAmplicon pipeline in a Linux environment. The first step is software installation. Bugbase is typically pre‐integrated within the EasyMicrobiome environment, but the original code needs to be updated to run. Generally, there are two options: the first is recommended, and the second is optional and only needs to be run once. The first method requires Git: git clone https://github.com/knights-lab/BugBase. The second method requires manual download by running the following code:

wget ‐c https://github.com/knights-lab/BugBase/archive/master.zip

mv master.zip BugBase.zip

unzip BugBase.zip

mv BugBase‐master/BugBase

cd BugBase

Install dependencies:

export BUGBASE_PATH = 'pwd'

export PATH = $PATH:'pwd'/bin

run.bugbase.r –h

Second step is preparing “biom format” input files based on Greengene OTU table (local analysis supports txt format without conversion) and mapping file (add # to the first line of metadata.txt). Run the following code:

cd ~/EasyAmplicon/result

biom convert ‐i gg/otutab.txt ‐o otutab_gg.biom ‐‐table‐type=“OTU table" ‐‐to‐json

sed '1s/^/#/' metadata.txt > MappingFile.txt

Local analysis with Bugbase.

export BUGBASE_PATH='pwd'

export PATH=$PATH:'pwd'/bin

run.bugbase.r ‐i otutab_gg.txt ‐m MappingFile.txt ‐c Group ‐o phenotype/

The “−i” indicates path to the OTU table (otutab_gg.txt), “‐m” indicates path to the mapping file (MappingFile.txt), “‐c” indicates column name in the mapping file containing group information (Group), and “‐o” indicates output directory for phenotype prediction results (phenotype/). The results of the analysis are shown in Figure 5A, under the heading Bugbase bacterial phenotype prediction.

Note: The output files and chart generated from the analysis mentioned above, including LEFSe, Picrust, FAPROTAX, and Bugbase, are shown in Figure 5A–D, respectively.

#### 6.5.5 Silme2 Random Forest/Adaboost

This section details the process of performing machine learning classification using Silme2 within the EasyAmplicon pipeline. Silme2 supports Random Forest and AdaBoost algorithms for microbiome analysis. The first step is software installation and dependencies. The instructions here are provided for reference. It is recommended to consult the Silme2 documentation for the latest installation and dependency procedures.

cd ~/software/# Replace with your desired download directory

wget https://github.com/swo/slime2/archive/master.zip

mv master.zip slime2.zip

unzip slime2.zip

mv slime2‐master/slime2

cp slime2/slime2.py ~/bin/# Replace with your desired bin directory

chmod +x ~/bin/slime2.py

sudo pip3 install ‐‐upgrade pip

sudo pip3 install pandas

sudo pip3 install sklearn

Further, run the Silme2 analysis under the QIIME 2 environment. Activate the QIIME 2 environment by running this code “conda activate qiime2‐2024.5” (Ensure you are using the correct QIIME 2 version). Navigate to the Silme2 script directory: “cd/mnt/d/EasyMicrobiome/script/slime2” (replace with your actual script path). For normalization, run this command: “./slime2.py otutab.txt design.txt ‐‐normalize ‐‐tag ab_e4 ab ‐n 10000.” The “otutab.txt” indicates the path to users OTU table in txt format. “design.txt” indicates the path to the design file with sample metadata and outcome variable. “‐‐normalize” indicates normalize data (recommended). “‐‐tag ab_e4” indicates the output tag for AdaBoost results (e.g., ab_e4 for AdaBoost with 10,000 iterations). “ab” indicates feature of interest for classification (replace with your actual feature name). “‐n 10000” indicates the number of iterations for AdaBoost (recommended to use millions for robust models). Subsequently, to use RandomForest and calculate 10,000 times in a short time (for robustness, recommend using millions of iterations, supports multi‐threading), run this code:

./slime2.py otutab.txt design.txt ‐‐normalize ‐‐tag rf_e4 rf ‐n 10000

#### 6.5.6 PICRUSt2 environment import and export

PICRUSt2 expands the capabilities of the original PICRUSt method [29] to predict the functional potential of a community based on marker gene sequencing profiles. The first step is to install PICRUSt2 (https://github.com/picrust/picrust2), for which several installation methods are available:

**Method 1:**

n=picrust2

conda create ‐n ${n} ‐c bioconda ‐c conda‐forge ${n}=2.3.0_b

conda activate ${n}

**Method 2:** Export the installation environment “cd ~/db/conda/.” Set the environment name “n=picrust2.” Then, run the following code to activate the picrust and compressed package.

conda activate ${n}

conda pack ‐n ${n} ‐o ${n}.tar.gz

**Method 3:** Import the installation environment, such as qiime2 and humann2 (including picurst), start by running code “n=picrust2” to set the environment name. Next, download the environment package with the following command:

wget ‐c ftp://download.nmdc.cn/tools/conda/${n}.tar.gz

Specify the installation directory and unzip the package.

condapath=~/miniconda3

mkdir ‐p ${condapath}/envs/${n}

tar ‐xvzf ${n}.tar.gz ‐C ${condapath}/envs/${n}

Finally, activate and initialize the environment by running this code:

source ${condapath}/envs/${n}/bin/activate

conda unpack

#### 6.5.7 PICRUSt2 function prediction

After activating the PICRUSt2 environment, run the following code step by step starting with Conda activation. To load the picrust2 environment, run the code “conda activate picrust2.” Then, run the following commands to create a directory named “picrsut2.”

wd = /mnt/c/EasyAmplicon/result/picrust2

mkdir ‐p ${wd} && cd ${wd}

Then, execute this command:

picrust2_pipeline.py ‐s../otus.fa ‐i../otutab.txt ‐o./out ‐p 8. This process utilizes OTU sequence file (../otus.fa), and OTU table (../otutab.txt). It may take approximately 12 min to complete. Then, add EC/KO/Pathway notes by executing the following code:

cd out

add_descriptions.py ‐i pathways_out/path_abun_unstrat.tsv.gz ‐m METACYC\

‐o pathways_out/path_abun_unstrat_descrip.tsv.gz

add_descriptions.py ‐i EC_metagenome_out/pred_metagenome_unstrat.tsv.gz ‐m EC\

‐o EC_metagenome_out/pred_metagenome_unstrat_descrip.tsv.gz

add_descriptions.py ‐i KO_metagenome_out/pred_metagenome_unstrat.tsv.gz ‐m KO\

‐o KO_metagenome_out/pred_metagenome_unstrat_descrip.tsv.gz

To merge KEGG by hierarchy, run the following code:

db=/mnt/c/EasyMicrobiome/

zcat KO_metagenome_out/pred_metagenome_unstrat.tsv.gz > KEGG.KO.txt

python3 ${db}/script/summarizeAbundance.py\

‐i KEGG.KO.txt

‐m ${db}/kegg/KO1‐4.txt\

‐c 2,3,4 ‐s ',+,+,' ‐n raw\

‐o KEGG

Finally, to count the number of features at each level, run the code wc ‐l KEGG*.

Note: This analysis is resource‐intensive and might require a server environment with sufficient memory (>16GB).

### 6.6 Analyses under QIIME 2 pipeline

EasyAmplicon seamlessly integrates with QIIME 2 [64], a popular bioinformatics platform for analyzing microbiome sequencing data. This section provides a basic guide for using QIIME 2 within the EasyAmplicon workflow. While QIIME 2 is primarily designed for Linux and Mac systems, Windows users can leverage the WSL or a Linux server environment.

Video tutorial link: https://youtu.be/PghGX9zXqm0

#### 6.6.1 QIIME 2 installation

EasyAmplicon provides clear instructions and potentially pre‐configured environments to manage QIIME 2 installation on your system (Figure 5E). To proceed with the qiime2, navigate to the EasyAmplicon master/qiime2 directory, open the “qiime2_pipeline” with Rstudio. Next, open a new terminal using Windows Subsystem for Linux (WSL) and activate the existing Conda environment by running the following code:

n=qiime2‐amplicon‐2024.5

wget ‐c https://data.qiime2.org/distro/amplicon/${n}‐py39‐linux‐conda.yml

conda env create ‐n ${n} ‐‐file ${n}‐py39‐linux‐conda.yml

conda pack ‐n ${n} ‐o ${n}.tar.gz

conda activate ${n}

Note: For Conda installation, refer to the ‘Downloading and Installing Conda’ section for detailed instructions.

#### 6.6.2 Downloading species annotation data

This protocol includes downloading two pre‐trained reference databases for species annotation (Figure 5E) including Silva 138 99% OTUs full‐length sequences and Greengenes2 2022.10 or later full‐length sequences. Users can download the desired database from the official QIIME 2 website. To download Silva 138 run this code:

wget ‐c https://data.qiime2.org/2024.2/common/silva-138-99-nb-classifier.qza

For Greengenes2, run the following code:

wget ‐c http://ftp.microbio.me/greengenes_release/2022.10/2022.10.backbone.full-length.nb.qza

#### 6.6.3 Training the classifier with a reference database

This section demonstrates how to train a custom classifier using a downloaded reference database, with Greengenes as an example. First, set up the working directory by running the following code:

wd= /mnt/c/EasyAmplicon/qiime2

mkdir ‐p $wd

cd $wd

To download the Greengenes reference database (a smaller 99% OTU database is also provided), run the following code:

wget ‐c ftp://greengenes.microbio.me/greengenes_release/gg_13_5/gg_13_8_otus.tar.gz

wget ‐c ftp://download.nmdc.cn/tools/amplicon/GreenGenes/gg_13_8_otus_99.tar.gz

mv gg_13_8_otus_99.tar.gz gg_13_8_otus.tar.gz

To extract the downloaded archive, run this code:

tar ‐zxvf gg_13_8_otus.tar.gz

To import the reference sequences from the ‘99_otus. fasta’ file within the ‘rep_set’ directory of the extracted archive ‘gg_13_8_otus’ into a QIIME artifact named 99_otus. qza, run the following code:

qiime tools import ‐‐type 'FeatureData[Sequence]' ‐‐input‐path gg_13_8_otus/rep_set/99_otus.fasta ‐‐output‐path 99_otus.qza

Then, to imports the species taxonomy information from the “99_OTU_taxonomy.txt” file within the taxonomy directory of the extracted archive “gg_13_8_otus” into a QIIME artifact named “ref‐taxonomy.qza,” run this code:

qiime tools import ‐‐type 'FeatureData[Taxonomy]' ‐‐input‐format HeaderlessTSVTaxonomyFormat ‐‐input‐path gg_13_8_otus/taxonomy/99_otu_taxonomy.txt ‐‐output‐path ref‐taxonomy.qza

To train the full‐length classifier named “classifier_gg_13_8_99.qza” (which may take 30 min), use the following code. The “‐‐i‐reference‐reads” flag specifies the imported reference sequences 99_otus.qza, and “‐‐i‐reference‐taxonomy” specifies the imported taxonomy information ref‐taxonomy.qza.

time qiime feature‐classifier fit‐classifier‐naive‐bayes ‐‐i‐reference‐reads 99_otus.qza ‐‐i‐reference‐taxonomy ref‐taxonomy.qza ‐‐o‐classifier classifier_gg_13_8_99.qza

To extract amplified segments based on the forward (AACMGGATTAGATACCCKG) and reverse (ACGTCATCCCCACCTTCC) primers (which may take 8 min), run the following code. The extracted sequences are stored in a QIIME artifact named “ref‐seqs. qza.” Remember to replace these primers with the actual primers used in the user's experiment.

time qiime feature‐classifier extract‐reads ‐‐i‐sequences 99_otus.qza ‐‐p‐f‐primer AACMGGATTAGATACCCKG ‐‐p‐r‐primer ACGTCATCCCCACCTTCC ‐‐o‐reads ref‐seqs.qza

To train the classifier using the extracted segments (which may take 7 min), run the following code:

time qiime feature‐classifier fit‐classifier‐naive‐bayes ‐‐i‐reference‐reads ref‐seqs.qza ‐‐i‐reference‐taxonomy ref‐taxonomy.qza ‐‐o‐classifier classifier_gg_13_8_99_V5‐V7.qza

#### 6.6.4 Basic workflow with Qiime2

Once all the software is successfully installed, users can begin basic analysis using the QIIME 2 pipeline (Figure 5F).

##### 6.6.4.1 Preparatory work

To start, first set up the working directory by running the following code:

wd= /mnt/c/Easyamplicon/qiime2/

mkdir ‐p ${wd}

cd ${wd}

Activate the QIIME2 environment (replace the qiime2 version with your specific version if you are using different one).

conda activate qiime2‐amplicon‐2024.5

Prepare sample metadata file (metadata.txt) and raw sequence data files (seq/*. fq.gz).

ln/mnt/c/EasyAmplicon/seq/* seq/

ln/mnt/c/EasyAmplicon/result/metadata.txt./

Generate a manifest file listing sample IDs, forward, and reverse FASTQ file paths based on your metadata.

awk 'NR==1{print "sample‐id\tforward‐absolute‐filepath\treverse‐ absolute‐filepath"} NR>1{print $1"\t"$PWD/seq/"$1"_1.fq.gz\t"$PWD/seq/"$1"_2.fq.gz"}' metadata.txt > manifest

head ‐n3 manifest

Import your data into QIIME2 as paired‐end Illumina sequences with Phred+33 quality scores.

qiime tools import ‐‐type 'SampleData[PairedEndSequencesWithQuality]' ‐‐input‐path manifest ‐‐output‐path demux.qza ‐‐input‐format PairedEndFastqManifestPhred33V2

##### 6.6.4.2 Generate feature tables and representative sequences

There are two main ways to generate feature tables and representative sequences in QIIME2:

**Method 1** (DADA2 (Optional)): This method performs denoising using DADA2, while it may be slower and more prone to errors compared to the recommended method.

time qiime dada2 denoise‐paired ‐‐i‐demultiplexed‐seqs demux.qza ‐‐p‐n‐threads 4 ‐‐p‐trim‐left‐f 29 ‐‐p‐trim‐left‐r 18 ‐‐p‐trunc‐len‐f 0 ‐‐p‐trunc‐len‐r 0 ‐‐o‐table dada2‐table.qza ‐‐o‐representative‐sequences dada2‐rep‐seqs.qza ‐‐o‐denoising‐stats denoising‐stats.qza

**Method 2** (external import): This is the recommended approach, allowing users to import pre‐generated feature tables (e.g., OTU table) and representative sequences from other software. To convert OTU table (otutab.txt) to biom format, run the following command:

biom convert ‐i otutab.txt ‐o otutab.biom ‐‐table‐type=“OTU table" ‐‐to‐json

To import the feature table and representative sequences into QIIME2, run this code:

qiime tools import ‐‐input‐path otutab.bi‐‐type 'FeatureTable[Frequency]' ‐‐input‐format BIOMV100Format ‐‐output‐path table.qza

To import the representative sequence, run the code given below:

qiime tools import ‐‐input‐path otus.fa ‐‐type 'FeatureData[Sequence]' ‐‐output‐path rep‐seqs.qza

Once imported, use these commands to generate summary statistics for the data:

qiime feature‐table summarize ‐‐i‐table table.qza ‐‐o‐visualization table.qzv ‐‐m‐sample‐metadata‐file metadata.txt

qiime feature‐table tabulate‐seqs ‐‐i‐data rep‐seqs.qza ‐‐o‐visualization rep‐seqs.qzv

##### 6.6.4.3 Alpha and beta diversity analysis

In this step, a phylogenetic tree is constructed to represent the evolutionary relationships between the microbial sequences in the data set. It takes the representative sequences “rep‐seqs.qza,” as input and generates several outputs, including aligned sequences “aligned‐rep‐seqs.qza,” masked aligned sequences “masked‐aligned‐rep‐seqs.qza” (containing only informative positions), unrooted phylogenetic tree “unrooted‐tree.qza,” rooted phylogenetic tree “rooted‐tree.qza.” Run the following command:

qiime phylogeny align‐to‐tree‐mafft‐fasttree ‐‐i‐sequences rep‐seqs.qza ‐‐o‐alignment aligned‐rep‐seqs.qza ‐‐o‐masked‐alignment masked‐aligned‐rep‐seqs.qza ‐‐o‐tree unrooted‐tree.qza ‐‐o‐rooted‐tree rooted‐tree.qza

Calculate core diversity metrics at a specific sampling depth (obtained from the feature table summary) by running this code:

qiime diversity core‐metrics‐phylogenetic ‐‐i‐phylogeny rooted‐tree.qza ‐‐i‐table table.qza ‐‐p‐sampling‐depth 7439 ‐‐m‐metadata‐file metadata.txt ‐‐output‐dir core‐metrics‐results

Analyze and visualize alpha diversity differences between groups defined in the metadata file and users can choose various alpha diversity indices (e.g., observed_features).

index=observed_features

qiime diversity alpha‐group‐significance ‐‐i‐alpha‐diversity core‐metrics‐results/${index}_vector.qza ‐‐m‐metadata‐file metadata.txt ‐‐o‐visualization core‐metrics‐results/${index}‐group‐significance.qzv

To generate alpha diversity rarefaction curves to visualize how diversity changes with sequencing depth, run this code:

qiime diversity alpha‐rarefaction ‐‐i‐table table.qza ‐‐i‐phylogeny rooted‐tree.qza ‐‐p‐max‐depth 10298 ‐‐m‐metadata‐file metadata.txt ‐‐o‐visualization alpha‐rarefaction.qzv

Analyze and visualize beta diversity differences between groups using a chosen distance metric (e.g., weighted_unifrac) and PERMANOVA tests.

distance=weighted_unifrac

column=Group

qiime diversity beta‐group‐significance

‐‐i‐distance‐matrix core‐metrics‐results/${distance}_distance_matrix.qza

‐‐m‐metadata‐file metadata.txt\

‐‐m‐metadata‐column ${column}\

‐‐o‐visualization core‐metrics‐results/${distance}‐${column}‐significance.qzv\

‐‐p‐pairwise

##### 6.6.4.4 Species composition analysis

This section guides users through classifying sequences using pre‐trained classifiers, such as the SILVA database [65].

time qiime feature‐classifier classify‐sklearn\

‐‐i‐classifier classifier_gg_13_8_99.qza

‐‐i‐reads rep‐seqs.qza\

‐‐o‐classification taxonomy.qza

To visualize the taxonomic composition of samples using QIIME2 visualizations, run the following code:

qiime metadata tabulate\

‐‐m‐input‐file taxonomy.qza

‐‐o‐visualization taxonomy.qzv

Display data with stacked bar charts:

qiime taxa barplot

‐‐i‐table table.qza\

‐‐i‐taxonomy taxonomy.qza\

‐‐m‐metadata‐file metadata.txt

‐‐o‐visualization taxa‐bar‐plots.qzv

##### 6.6.4.5 Differential feature analysis using ANCOM

To add pseudo‐counts to feature table for robust statistical analysis, run the code given below:

qiime composition add‐pseudocount

‐‐i‐table table.qza\

‐‐o‐composition‐table comp‐table.qza

To identify the differentially abundant features between groups using ANCOM, run the following command:

column=Group

time qiime composition ancom\

‐‐i‐table comp‐table.qza\

‐‐m‐metadata‐file metadata.txt

‐‐m‐metadata‐column ${column}\

‐‐o‐visualization ancom‐${column}.qzv

Optionally, perform differential abundance analysis at a higher taxonomic level (e.g., genus) by collapsing the feature table. Merging can be performed by executing the given code (e.g., at genus level):

qiime taxa collapse\

‐‐i‐table table.qza\

‐‐i‐taxonomy taxonomy.qza\

‐‐p‐level 6\

‐‐o‐collapsed‐table table‐l6.qza

Format the feature table and add pseudo‐counts via running this code:

qiime composition add‐pseudocount\

‐‐i‐table table‐l6.qza\

‐‐o‐composition‐table comp‐table‐l6.qza

To calculate differential genera, run the following code and specify the type of grouping for comparison:

qiime composition ancom\

‐‐i‐table comp‐table‐l6.qza\

‐‐m‐metadata‐file metadata.txt\

‐‐m‐metadata‐column ${column}

‐‐o‐visualization ancom‐l6‐${column}.qzv

### 6.7 Anticipated results

The EasyAmplicon protocol includes scripts and explanations for each step, with references to external resources, enabling ease of use and adaptation. It supports batch processing of diversity indices, automates calculations, and generates visualizations like boxplots, histograms, dilution curves, and Venn diagrams for alpha diversity. Beta diversity is analyzed using distance matrices and PCoA plot, etc. Functional predictions and species compositions are examined using reference databases, stacked bar charts, chord diagrams, and tree maps. LEfSe identifies differentially abundant features, while PICRUSt2 predicts functional pathways. FAPROTAX analyzes OTU roles in elemental cycling. The comprehensive analysis using EasyAmplicon pipeline offers valuable insights into the composition and diversity of the microbiome sample (plant, animal, soil, environment), paving the way for further exploration of any host‐microbe interactions. This detailed protocol is intended to empower researchers across various fields to delve into microbiome research with greater ease and confidence, ultimately broadening the scope and impact of microbial community studies. While primarily designed for second‐generation sequencing data, future updates aim to introduce additional functions and improve compatibility with third‐generation platforms.

## 7 CONCLUSION

The EasyAmplicon protocol presented here provides a comprehensive, detailed guide for researchers to perform amplicon analysis efficiently and effectively. By integrating multiple popular bioinformatics tools into a streamlined workflow, it significantly lowers the technical barriers typically associated with high‐throughput data analysis. This protocol meticulously explains each step in detail, from data preprocessing and diversity analysis to advanced visualization techniques, ensuring that users can follow along without extensive bioinformatics expertise.

## AUTHOR CONTRIBUTIONS

**Salsabeel Yousuf**: Writing—original draft; formal analysis; validation; writing—review and editing; visualization. **Hao Luo**: Validation; writing—review and editing; visualization; formal analysis. **Meiyin Zeng**: Validation; writing—review and editing; visualization; formal analysis. **Lei Chen**: Validation; writing—review and editing; formal analysis. **Tengfei Ma**: Validation; writing—review and editing; formal analysis. **Xiaofang Li**: Writing—review and editing; formal analysis. **Maosheng Zheng**: Writing—review and editing; formal analysis. Xin Zhou: Writing—review and editing; formal analysis. **Liang Chen**: Validation; writing—review and editing. **Jiao Xi**: Validation; writing—review and editing. **Hongye Lu**: Validation; writing—review and editing. **Huiluo Cao**: Validation; writing—review and editing. **Xiaoya Ma**: Validation; writing—review and editing. **Bian Bian**: Validation; writing – review and editing. **Pengfan Zhang**: Validation; writing – review and editing. **Jiqiu Wu**: Validation; writing – review and editing. **Ren‐You Gan**: Validation; writing—review and editing. **Baolei Jia**: Validation; writing–review and editing. **Linyang Sun**: Validation; writing—review and editing; formal analysis. **Zhicheng Ju**: Validation; writing – review and editing; formal analysis. **Yunyun Gao**: Validation; writing—review and editing. **Waqar Afzal Malik**: Writing—review and editing; writing—original draft; validation. **Chuang Ma**: Validation; writing—review and editing. **Hujie Lyu**: Validation; writing—review and editing. Yahui Li: Validation; writing—review and editing. **Hui‐yu Hou**: Validation; writing—review and editing. **Yuanping Zhou**: Validation; writing—review and editing. **Defeng Bai**: Validation; writing—review and editing. **Yao Wang**: Validation; writing—review and editing. **Haifei Yang**: Validation; writing—review and editing. **Jiani Xun**: Validation; writing—review and editing. **Shengda Du**: Validation; writing—review and editing. **Tianyuan Zhang**: Validation; writing—review and editing. **Xiulin Wan**: Validation; writing—review and editing. **Kai Peng**: Validation; writing—review and editing. **Shanshan Xu**: Validation; writing—review and editing. **Tao Wen**: Validation; writing—review and editing; visualization; formal analysis; software. **Tong Chen**: Validation; writing—review and editing; visualization; formal analysis; software; Data curation; methodology. **Yong‐xin Liu**: Developed pipeline; Data curation; conceptualization; funding acquisition; writing— review and editing; project administration; resources; supervision; methodology; validation; visualization; investigation; software; formal analysis. All authors have read the final manuscript and approved it for publication.

## CONFLICT OF INTEREST STATEMENT

The authors declare no conflicts of interest.

## ETHICS STATEMENT

No animals or humans were involved in this study.

## Supporting information

The online version contains supplementary figures and tables available.

RELATED VIDEOS
11 Amplicon software installations: https://youtu.be/ZFrqi5P2i8M
22 Sequencing Data into Features Table part 1: https://www.youtube.com/watch?v=9ORamd84hUc
22 Sequencing Data into Features Table part 2: https://youtu.be/R7wj3D9B6xU
23 Species Diversity Analysis: https://youtu.be/n13M8p_IrXk
24 Differential Analysis 1‐R: https://youtu.be/FsdYNQ5DtHg
24 Differential Analysis 2‐STAMP: https://youtu.be/PptGLAI93eE
24 Differential Analysis 3‐LEFSe: https://youtu.be/VXJvdidyFlU
31 Feature Prediction 2‐ FAPRPTAX: https://youtu.be/kAiMq08mLNs
31 Feature Prediction 1‐Picrust: https://youtu.be/bq9IkEJVAoQ
31 Feature Prediction 3‐Phenotype Bugbase: https://youtu.be/OvkZxjOjwmE
33 Phylogenetic construction and Beatification of Evolutionary Tree: https://youtu.be/c3cM-zwbChs
25 Qiime2 amplicon analysis process: https://youtu.be/PghGX9zXqm0.

Figure S1. Overview of the ImageGP platform for microbial data analysis and visualization.

## Data Availability

Data sharing is not applicable to this article as no new data were created or analyzed in this study. The computational scripts for the EasyAmplicon v1.0 data analysis pipeline are freely accessible on GitHub at https://github.com/YongxinLiu/EasyAmplicon. The EasyAmplicon web server is also freely available and regularly updated with the latest versions of the software and databases. Users are encouraged to refer to the GitHub repository for updated links related to the EasyAmplicon process. Supplementary materials (figures, scripts, graphical abstract, slides, videos, Chinese translated version and updated materials) may be found in the online DOI or iMeta Science http://www.imeta.science/imetaomics/. Correspondence and requests for materials should be addressed to Yong‐Xin Liu.
