# Supplementary material for: Unveiling microbial communities with EasyAmplicon: A user‐centric guide to perform amplicon sequencing data analysis
Source: IMetaOmics. 2024 Nov 20;1(2):e42. doi: 10.1002/imo2.42 (PMC12806499; doi:10.1002/imo2.42)
Supplement: Supplementary file 1 — RELATED VIDEOS 11 Amplicon software installations: https://youtu.be/ZFrqi5P2i8M 22 Sequencing Data into Features Table part 1: https://www.youtube.com/watch?v=9ORamd84hUc 22 Sequencing Data into Features Table part 2: https://youtu.be/R7wj3D9B6xU 23 Species Diversity Analysis: https://youtu.be/n13M8p_IrXk 24 Differential Analysis 1‐R: https://youtu.be/FsdYNQ5DtHg 24 Differential Analysis 2‐STAMP: https://youtu.be/PptGLAI93eE 24 Differential Analysis 3‐LEFSe: https://youtu.be/VXJvdidyFlU 31 Feature Prediction 2‐ FAPRPTAX: https://youtu.be/kAiMq08mLNs 31 Feature Prediction 1‐Picrust: https://youtu.be/bq9IkEJVAoQ 31 Feature Prediction 3‐Phenotype Bugbase: https://youtu.be/OvkZxjOjwmE 33 Phylogenetic construction and Beatification of Evolutionary Tree: https://youtu.be/c3cM-zwbChs 25 Qiime2 amplicon analysis process: https://youtu.be/PghGX9zXqm0. [file IMO2-1-e42-s002.docx]

# Supporting information to

**Unveiling microbial communities with EasyAmplicon: A user-centric guide to perform amplicon sequencing data analysis**

**Running title:** A pipeline for comprehensive amplicon sequencing data analysis

Salsabeel Yousuf^1#^, Hao Luo^1#^, Meiyin Zeng^1#^, Lei Chen^2^, Tengfei Ma^3^, Xiaofang Li^4^, Maosheng Zheng^5^, Xin Zhou^6^, Liang Chen^7^, Jiao Xi^8^, Hongye Lu^9^, Huiluo Cao^10^, Xiaoya Ma^11,12^, Bian Bian^13^, Pengfan Zhang^14^, Jiqiu Wu^15,16^, Renyou Gan^17^, Baolei Jia^18^, Linyang Sun^19^, Zhicheng Ju^20^, Yunyun Gao^1^, Waqar Afzal Malik^1^, Chuang Ma^1,21^, Hujie Lyu^22^, Yahui Li^1^, Huiyu Hou^1^, Yuanping Zhou^1,23^, Defeng Bai^1^, Yao Wang^1^, Haifei Yang^1,24^, Jiani Xun^1^, Shengda Du^1,25^, Tianyuan Zhang^1^, Xiulin Wan^1,26^, Kai Peng^27^, Shanshan Xu^28^, Tao Wen^29^, Tong Chen^30^, Yongxin Liu^1^**^*^**

^1^Genome Analysis Laboratory of the Ministry of Agriculture and Rural Affairs, Agricultural Genomics Institute at Shenzhen, Chinese Academy of Agricultural Sciences, Shenzhen, Guangdong 518120, China

^2^Department of Vascular Surgery, Fu Xing Hospital, Capital Medical University, Beijing 100038, China

^3^State Key Laboratory of Herbage Improvement and Grassland Agro-Ecosystems, Centre for Grassland Microbiome, College of Pastoral Agriculture Science and Technology, Lanzhou University, Lanzhou, Gansu 730000, China

^4^Centre for Agricultural Resources Research, Institute of Genetics and Developmental Biology, Chinese Academy of Sciences, Shijiazhuang, Hebei 050021, China

^5^College of Environmental Science and Engineering, North China Electric Power University, Beijing 102206, China

^6^State Key Laboratory of Mycology, Institute of Microbiology, Chinese Academy of Sciences, Beijing 100101, China

^7^Biomedical Innovation Center and Beijing Key Laboratory for Therapeutic Cancer Vaccines, Beijing Shijitan Hospital, Capital Medical University, Beijing 100038, China

^8^College of Natural Resources and Environment, Northwest A&F University, Yangling, Shaanxi 712100, China

^9^Stomatology Hospital, School of Stomatology, Zhejiang University School of Medicine, Zhejiang Provincial Clinical Research Center for Oral Diseases, Key Laboratory of Oral Biomedical Research of Zhejiang Province, Cancer Center of Zhejiang University, Engineering Research Center of Oral Biomaterials and Devices of Zhejiang Province, Hangzhou, Zhejiang 310016, China

^10^Department of Microbiology, University of Hong Kong, Hong Kong 999077, China

^11^Center for Energy Metabolism and Reproduction, Institute of Biomedicine and Biotechnology, Shenzhen Institute of Advanced Technology, Chinese Academy of Sciences, Shenzhen, Guangdong 518055, China

^12^Department of Cardiology, Shenzhen Guangming District People’s Hospital, Shenzhen, Guangdong 518107, China

^13^Department of Computational Biology and Medical Sciences, Graduate School of Frontier Sciences, The University of Tokyo, Kashiwa, Chiba 277-8561, Japan

^14^Innovative Genomics Institute, University of California, Berkeley 94720, USA

^15^APC Microbiome Institute, University College Cork, Cork T12 K8AF, Ireland

^16^Department of Genetics, University Medical Center Groningen, University of Groningen, Groningen 9713 GZ, The Netherlands

^17^Department of Food Science and Nutrition, Faculty of Science, The Hong Kong Polytechnic University, Kowloon, Hong Kong 999077, China

^18^Xianghu Laboratory, Hangzhou, Zhejiang 311231, China

^19^Faculty of Biological & Environmental Sciences, University of Helsinki, Helsinki 00014, Finland

^20^Department of Ocean Science, The Hong Kong University of Science and Technology, Hongkong 999077, China

^21^School of Horticulture, Anhui Agricultural University, Hefei, Anhui 230036, China

^22^Department of Life Sciences, Imperial College of London, London SW7 2AZ, United Kingdom

^23^Guangdong Provincial Key Laboratory of Medical Molecular Diagnostics, The First Dongguan Affiliated Hospital, College of Basic Medicine, Guangdong Medical University, Dongguan, Guangdong 523808, China

^24^College of Life Sciences, Qingdao Agricultural University, Qingdao, Shandong 266109, China

^25^College of Life Sciences, Northwest A&F University, Yangling, Shaanxi 712100, China

^26^Guangdong Hybribio Biotech Co., Ltd., Chaozhou, Guangdong 521000, China

^27^Jiangsu Co-Innovation Center for Prevention and Control of Important Animal Infectious Diseases and Zoonoses, College of Veterinary Medicine, Yangzhou University, Yangzhou, Jiangsu 225009, China

^28^School of Food and Biological Engineering, Hefei University of Technology, Hefei, Anhui 230009, China

^29^Jiangsu Provincial Key Lab for Solid Organic Waste Utilization, Key Lab of Organic-Based Fertilizers of China, Jiangsu Collaborative Innovation Center for Solid Organic Wastes, Educational Ministry Engineering Center of Resource-Saving Fertilizers, Nanjing Agricultural University, Nanjing, Jiangsu 210095, China

^30^State Key Laboratory for Quality Ensurance and Sustainable Use of Dao-di Herbs, National Resource Center for Chinese Materia Medica, China Academy of Chinese Medical Sciences, Beijing 100000, China

^#^These authors contributed equally: Salsabeel Yousuf, Hao Luo, Meiyin Zeng

**^*^Correspondence**: [liuyongxin@caas.cn](mailto:liuyongxin@caas.cn) (Yongxin Liu)





**Figure S1. Overview of the ImageGP platform for microbial data analysis and visualization.** (A) ImageGP offers a range of visualization tools, including bar plots, heatmaps, and PCoA plots, which facilitate the efficient analysis of microbial data by revealing insights into community composition, diversity, and functional profiles. (B) The LEfSe module identifies differentially abundant microbial taxa between groups and discovers key taxa associated with specific conditions. (C) The PICRUSt module predicts the functional capabilities of microbial communities based on amplicon sequencing, offering results across various functional levels. (D) FAROTAX provides taxonomic classification and abundance analysis, allowing users to classify microbial communities to various taxonomic levels. (E) The BugBase module predicts phenotypic traits based on 16S rRNA gene data, providing insights into microbial community ecology.

**Related Videos**

11 Amplicon software installations: <https://youtu.be/ZFrqi5P2i8M>

22 Sequencing Data into Features Table part 1: <https://www.youtube.com/watch?v=9ORamd84hUc>

22 Sequencing Data into Features Table part 2: <https://youtu.be/R7wj3D9B6xU>

23 Species diversity Analysis: <https://youtu.be/n13M8p_IrXk>

24 Differential Analysis 1-R: <https://youtu.be/FsdYNQ5DtHg>

24 Differential Analysis 2-STAMP: <https://youtu.be/PptGLAI93eE>

24 Differential Analysis 3-LEFSe: <https://youtu.be/VXJvdidyFlU>

31 Feature Prediction 2- FAPRPTAX: <https://youtu.be/kAiMq08mLNs>

31 Feature Prediction 1-Picrust: <https://youtu.be/bq9IkEJVAoQ>

31 Feature Prediction 3-Phenotype Bugbase: <https://youtu.be/OvkZxjOjwmE>

33 Phylogenetic construction and Beatification of Evolutionary Tree: <https://youtu.be/c3cM-zwbChs>

25 Qiime2 amplicon analysis process: <https://youtu.be/PghGX9zXqm0>
