# Supplementary figures and images for: Unveiling microbial communities with EasyAmplicon: A user‐centric guide to perform amplicon sequencing data analysis
Source: IMetaOmics. 2024 Nov 20;1(2):e42. doi: 10.1002/imo2.42 (PMC12806499; doi:10.1002/imo2.42)

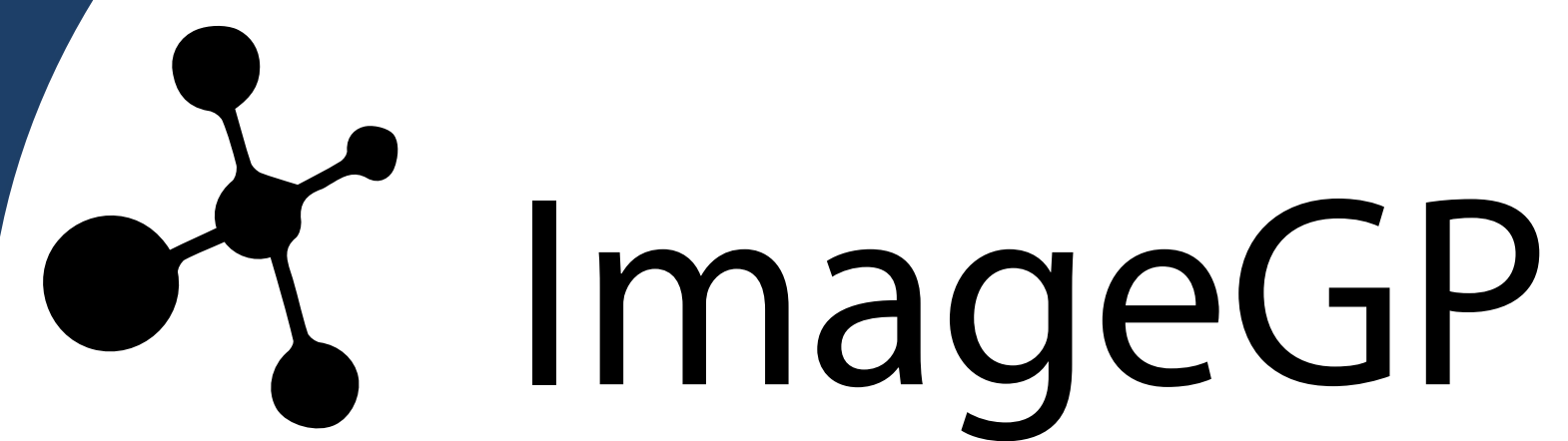

(A)

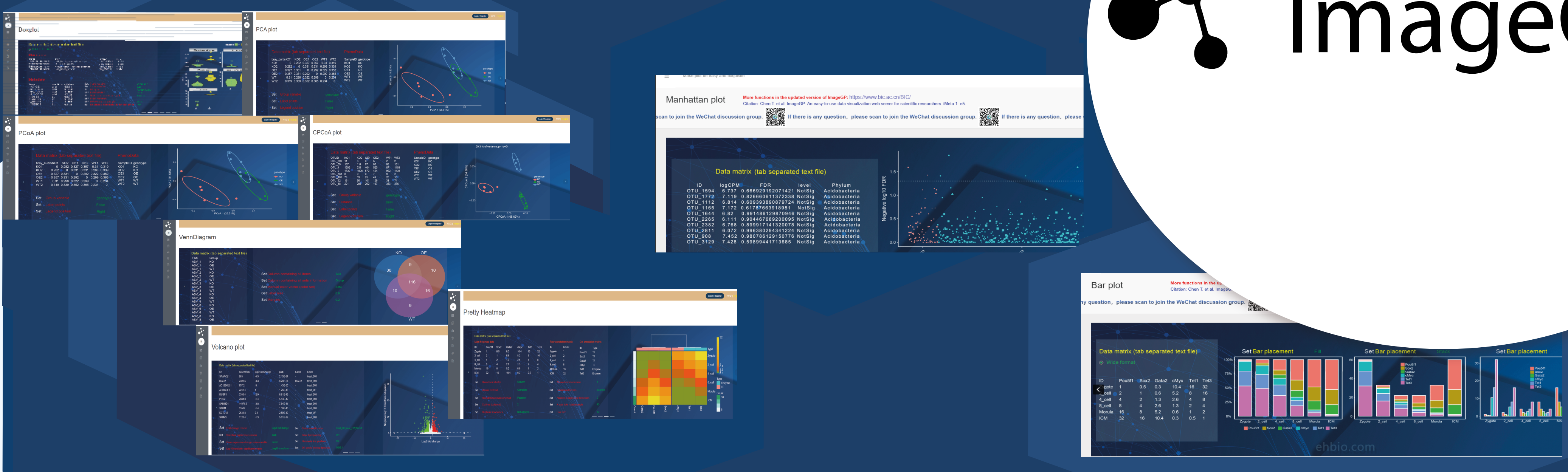

(B)

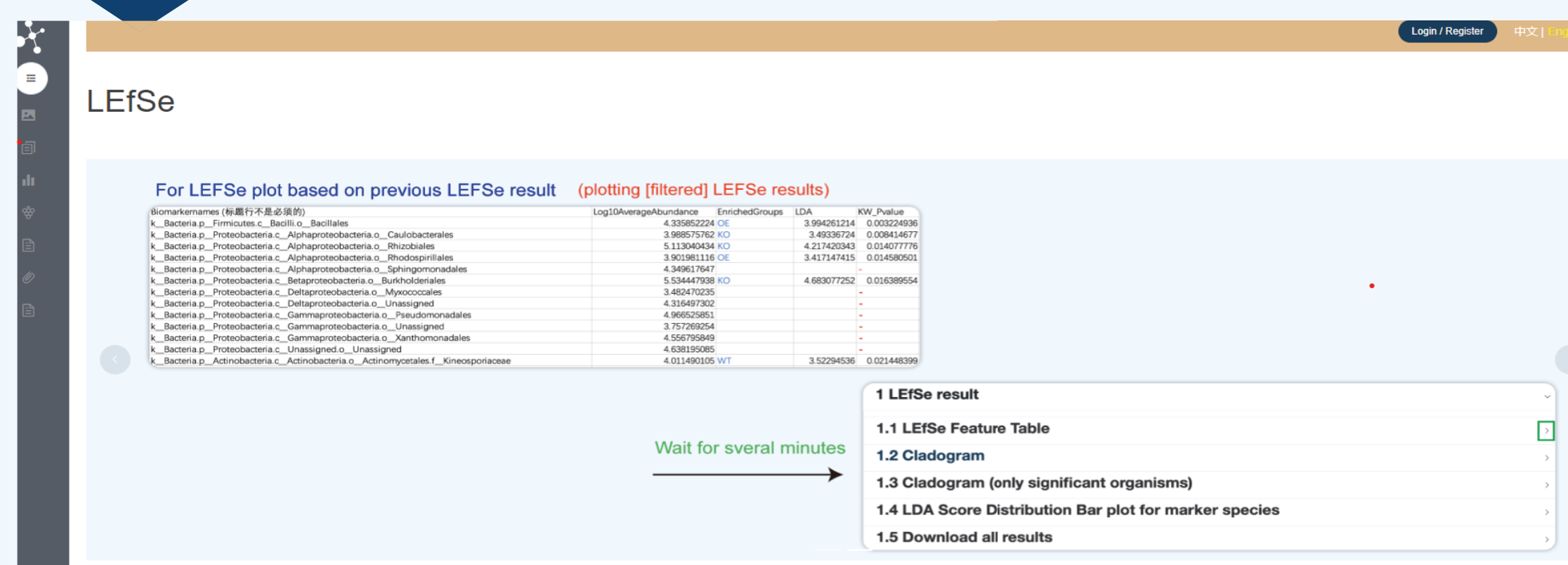

(C)

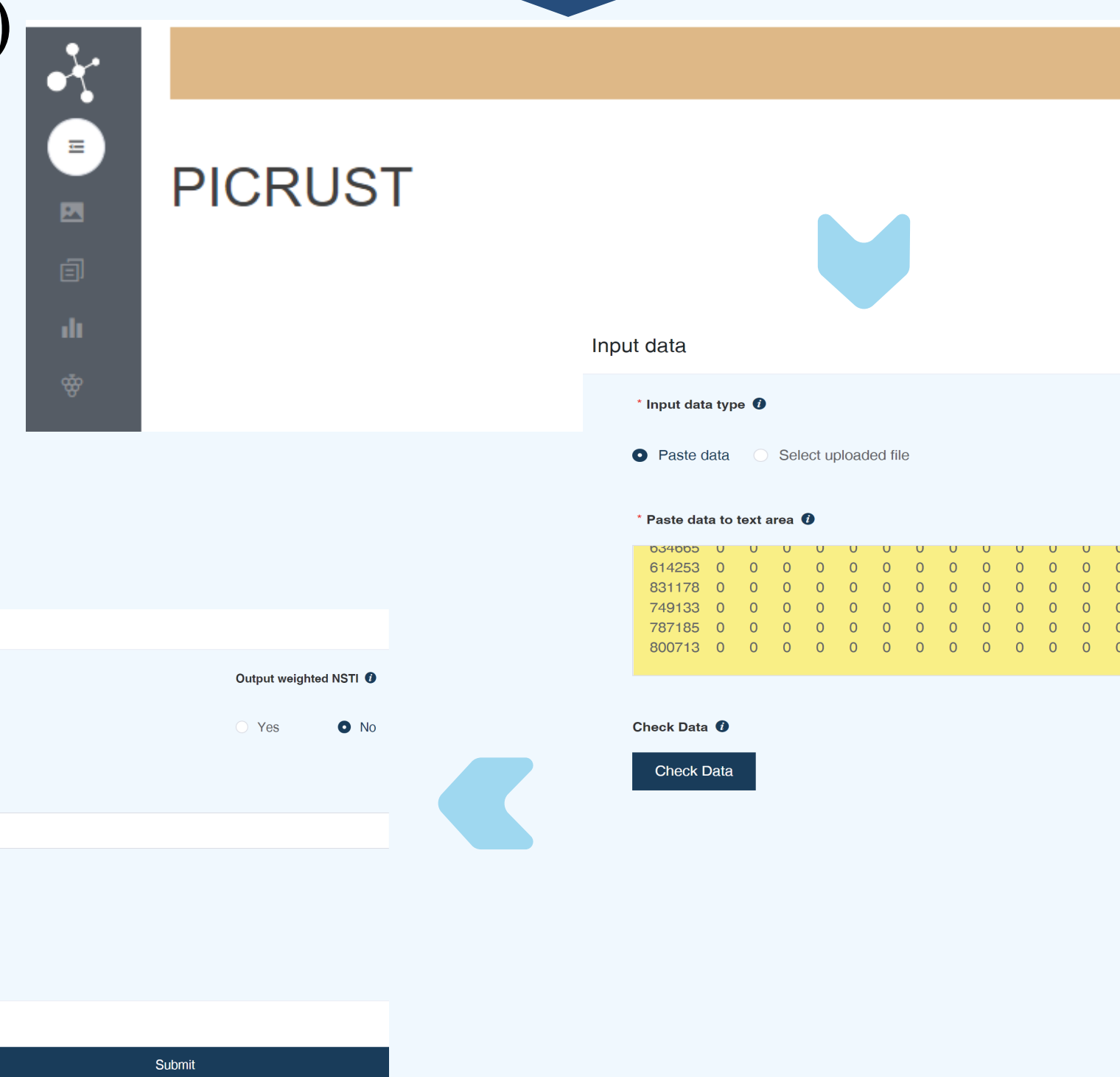

(D)

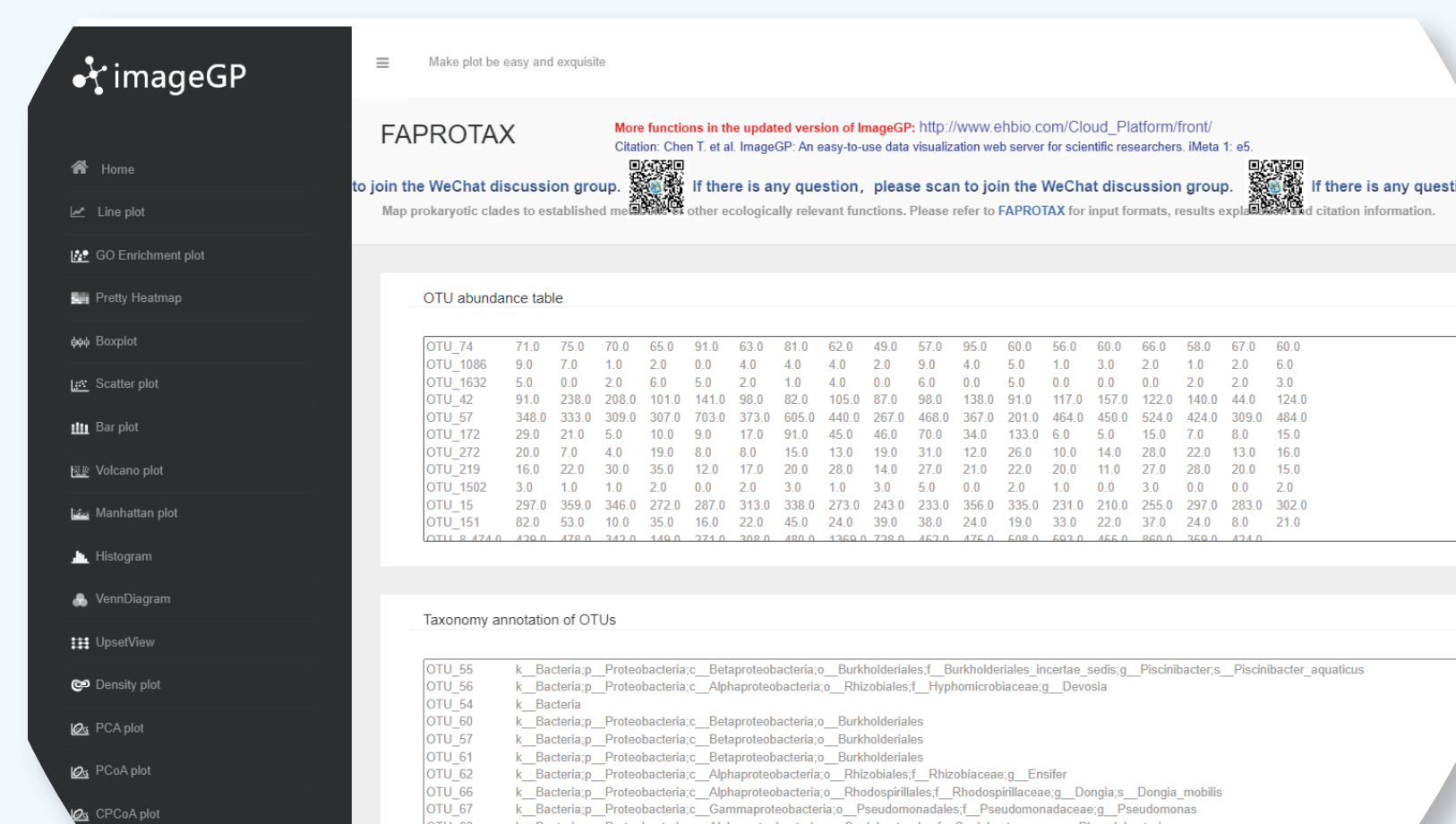

(E)

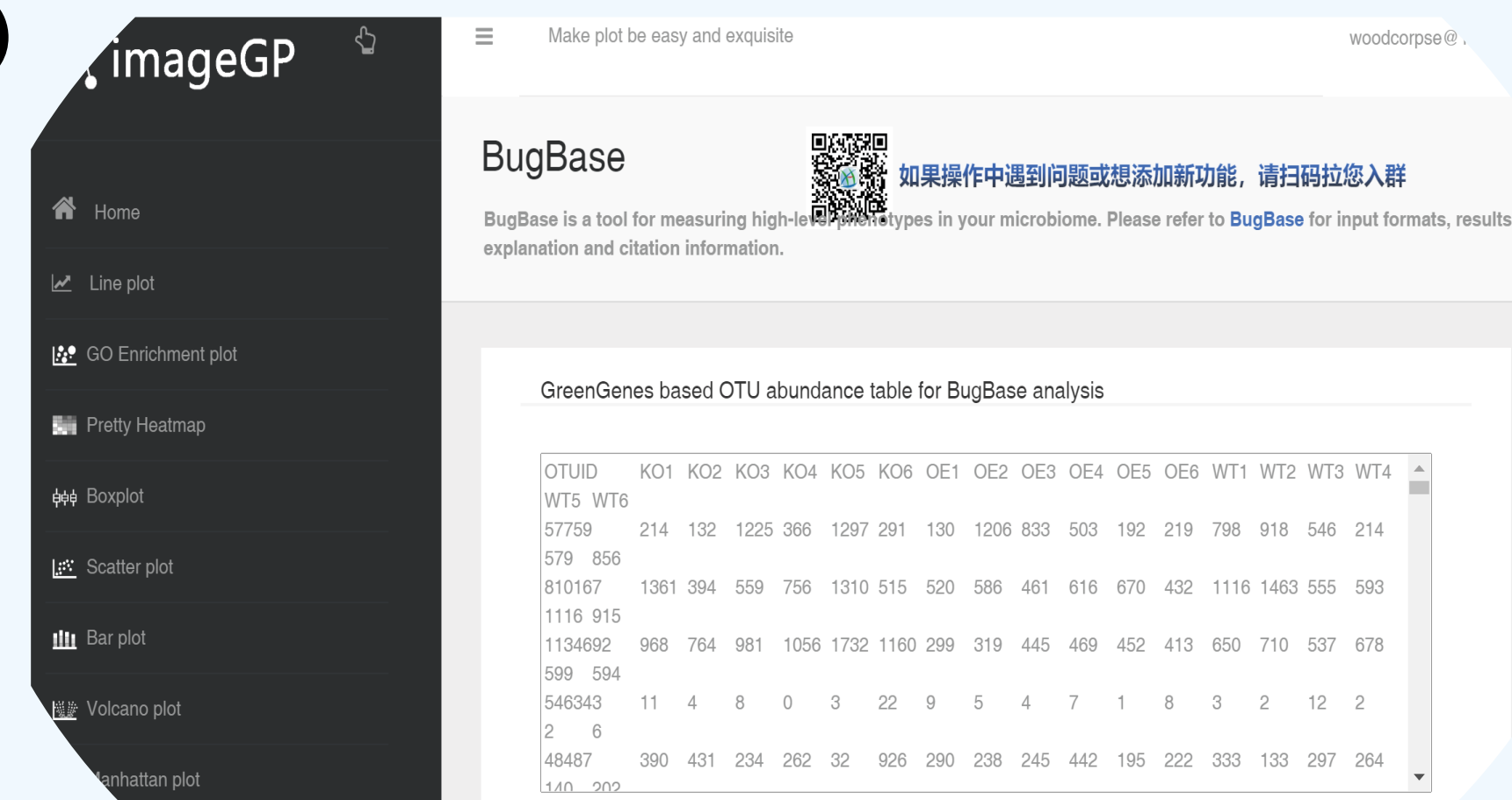

Supplement: Supplementary file 2 — Figure S1. Overview of the ImageGP platform for microbial data analysis and visualization. [file IMO2-1-e42-s001.pdf]
